# National survey evaluating the introduction of new and alternative staffing models in intensive care (SEISMIC-R) in the UK

**DOI:** 10.1136/bmjopen-2024-088233

**Published:** 2025-04-10

**Authors:** Rebecca Hadley, Burcu Dogan, Niamh Wood, Niamh Bohnacker, Paul R Mouncey, Natalie Pattison, SEISMIC-R investigator group, Peter Griffiths

**Affiliations:** 1 University of Hertfordshire, Hatfield, UK; 2 Intensive Care National Audit and Research Centre, London, UK; 3 Imperial College Healthcare NHS Trust, London, UK; 4 Clinical Trials Unit, ICNARC, London, UK; 5 School of Health and Social Work, University of Hertfordshire, Hatfield, UK; 6 East and North Hertfordshire NHS Trust, Stevenage, UK

**Keywords:** Adult intensive & critical care, Surveys and Questionnaires, Health policy, Nurses, Rationing

## Abstract

**Objective:**

To report on the findings from a national survey of UK intensive care units (ICUs) exploring nurse staffing models currently in use and changes since COVID-19.

**Design:**

A survey was designed and distributed using a web-based platform to senior unit leads via Intensive care national audit & research centre contacts.

**Participants:**

Senior nurses representing the 331 National Health Service adult ICUs across the UK (across 231 hospitals/155 trusts), including the Channel Islands and Isle of Man.

**Outcome measures:**

A 15-item survey.

**Results:**

A total of 196 survey responses representing 300 units, majority general and single units, resulting in a 90.6% unit-level response rate. ICU unit characteristics included the average number of total, level 3 and level 2 critical care beds of 26.36 (SD=21.48), 15.67 (SD=15.33) and 10.96 (SD=8.86), respectively. Most units reported nurse to patient ratios compliant with national guidelines and service specifications. Post-COVID-19 changes to ICU nurse staffing establishments were reported by 44% respondents, including increases in non-registered staff. However, limited data were provided regarding decision-making around and changes to bedside allocation of nurses since COVID-19.

**Conclusions:**

Increased numbers and use of non-registered staff within the ICU is indicative of an alternative staffing model to address nursing shortages. However, more research is needed to understand how this staffing group is being used compared with, and alongside, registered nurses.

**Trial registration number:**

Clinicaltrials.gov: NCT05917574.

**STRENGTHS AND LIMITATIONS OF THIS STUDY**

- Critical care is a complex area, predominantly staffed by nurses, but the models used in practice can be highly variable.
- This is the first survey to detail nursing models in use in the UK critical care units to provide an outline of possible areas for building capacity in the nursing workforce.
- Strengths of the study include a high response rate, with a known denominator based on comprehensive survey dissemination, extensive pilot and refinement and potential for replication.
- The inclusion of open and closed questions provided quantitative and rich qualitative data to support understanding of how models are used in practice.
- Potential limitations include piloting and identification of site respondents, which created some overlap and duplication of data, requiring de-duplication.

## Introduction

 Staffing critical care with adequate numbers of skilled nurses remains a global challenge.^1^ The UK has one of the lowest numbers of nurses per capita in Europe (8.7 per 1000 inhabitants), according to the international Organisation for Economic Co-operation and Development data^2^ and, historically, one of the lowest critical care bed numbers per 100 000 population.^3^ Despite the expansion of critical care bed numbers in the UK over the past decade of 13%^4^ and a yearly increase in intensive care unit (ICU) demand of 4%,^5^ national surveys have indicated a decrease in the number of registered nurses (RNs) currently employed in ICUs across England, Wales and Northern Ireland between 2017 and 2019,^6^ with turnover reaching 42% in some areas of the UK.^5^ The English National Health Service (NHS) specification determines nurse staffing ratios for ICUs, with a minimum of one RN providing direct care for one level 3 patient (highest acuity, based on organ failure) and a ratio of 1:2 for level 2 patients^7 8^ (see Methods section for levels of care descriptors). This national critical care commissioning guidance^7^ references professional body guidance,^9^ providing a blueprint for how services, including staffing models, should be organised. ICU capacity is almost entirely contingent on nurse numbers, especially during situations like pandemic scenarios.^10^ However, shifting workforce characteristics, such as dilution of skill mix and nurse: patient ratios and reduced staff availability,^5 11^ and rapid expansion of ICU capacity during the COVID-19 pandemic have led to an increased interest in and use of alternative nurse staffing models. Alternative models have included adjusted nurse to patient ratios as NHS trusts have struggled to meet service specifications in the face of increasing demand,^6^ particularly during COVID-19.^12^ The need for models that permit local variation and allow reporting against skill mix and staffing numbers has been highlighted across England.^13^

The Study to Evaluate the Introduction of new and alternative Staffing Models in Intensive Care study (SEISMIC) (NIHR ref: 200100)^13^,^15^ was the smaller-scale precursor study involving interviews and focus groups to understand staffing and to see how feasible data capture would be for the main study: *A Study to Evaluate the Introduction of new Staffing Models in Intensive Care: a Realist investigation (SEISMIC-R), NIHR reference: 135168*. SEISMIC took place during the pandemic and is distinct from this current study, SEISMIC-R. The survey forms part of this larger realist study which aims to explore the impact of the different staffing models being used in ICUs across the UK. Realist evaluation studies aim to provide programme theories to explain changes, outlining what works, for whom and in what circumstances.^16^ Here, we report the first phase of the study reported. Our earlier SEISMIC study sought to evaluate the introduction of alternative nurse staffing models in ICU on staff and patient outcomes, reporting on evidence to suggest that increased staff workload is associated with increased mortality and increased hospital-acquired infections.^14^

While studies evidence the impact of nurse staffing on patient outcomes, few have clearly outlined the characteristics constituting ‘nurse staffing’^14^ such as the number of registered and non-RNs in post, nurse to patient ratios, proportion of critical care qualified nurses and nurse allocation models. Critical care encompasses ICUs and high-dependency units, but for this study, we are focusing on ICUs providing the highest level of care (level 3 care), exploring the impact of different models on staff and patient outcomes, particularly within an ICU setting. Specifically, we report here on the results of a national survey of ICUs intended to establish the organisation of nurse staffing models, to provide an understanding of models in use and any changes to staffing models and practices since COVID-19.

## Methods

### Survey development and piloting

A national survey was developed with the aim of exploring current staffing models in use, changes since the COVID-19 pandemic, daily and total nurse staffing establishments, changes to establishments since COVID-19 and suggestions for alternative staffing models. The expected outcome was an understanding of the different staffing models being used. The survey was initially piloted with 11 Critical Care National Nurse Network Leads (CC3N, representing the 15 regional formalised NHS critical care networks in England) for content/face validity across seven items. Wording was then refined, and it was further piloted in 54 ICUs across England (convenience sampling, sent out across most critical care networks) via the National Nurse Network Leads in preparation for the main survey. A further eight items were added to the survey to address comprehensibility and the research questions, with further testing via the UK Critical Care Nursing Alliance leads, representing all critical care nursing organisations/critical care nurse professional bodies in the UK (n=8) to also address consequences of staffing models in use (nurse to patient ratios and nursing care structures) and pose open questions about staffing. Our patient and public involvement partners (SEISMIC-R study co-investigators) also reviewed the study, contributing to both design and content. The final 15-item survey (26 items with subquestions) (online supplemental file 2) was sent to leads of all 331 critical care units across the UK from August to December 2023.

### Sample

The target sample was the most senior nursing representative from each of the NHS (state-funded, not private) adult ICUs across the UK, including mixed ICUs/high dependency units (HDUs), general ICUs and single/mixed specialty units. Units were identified using the Intensive care national audit & research centre’s (ICNARC’s) Case Mix Programme—the national audit of patient outcomes from adult ICUs in the UK, Channel Islands and Isle of Man, with 100% coverage of adult general ICUs and most specialist units, making it highly representative of the operations within UK ICUs. This covered both level 1, 2 and 3 patients. Level 1 is enhanced care, for patients requiring a higher level of monitoring but not critical care; level 2 care is for those needing two or more basic organ system monitoring/support, single organ support at an advanced level (other than advanced respiratory support), long-term advanced respiratory support or high levels of nursing dependency not able to be provided in a level 1 area. Level 3 care involves advanced respiratory support or monitoring/support for two or more organ systems at an advanced level. It also includes level 2 patients with delirium/agitation or those with chronic impairment of at least one organ that restricts daily activities (comorbidity) and who require support for an acute reversible failure of another organ system.^8^ ICNARC is commissioned by the NHS and UK government to collate a minimum set of patient outcome data from all adult ICUs in England, Wales and Northern Ireland. The Scottish Intensive Care Society Audit Group was used to identify eligible Scottish units. Private ICUs, paediatric ICUs and HDUs were excluded from the sample, resulting in a target sample of 331 units (across 231 hospitals/155 trusts). NHS trusts frequently comprise several hospitals and several ICUs, which may be managed differently or independently even within the same NHS Trust.

### Patient and public involvement

Two patient and public involvement (PPI) co-investigators were involved from study inception, in survey design and review and throughout data collection and analysis, attending weekly study meetings. In addition to this, a realist advisory group (also including PPI members) contributed to the study development, including the survey.

### Study procedures

An email was sent to English, Welsh, Northern Irish and Scottish unit contacts via ICNARC requesting senior nurse lead contact for ICU (Band 8B/8C level, that is, a very senior grade of nurse such as matron, ICU head nurse or ICU lead nurse), ensuring the most appropriately qualified person completed it to maximise data accuracy. Sites were then sent an individualised link to the survey to prevent multiple responses and ensure that only authorised people had access. Hospitals with multiple critical care units were asked to complete one survey for all units, detailing unit staffing for each unit within that hospital. Where the lead nurse was delegated to hospital (ICU) leads, we analysed results for each, as above. We looked at each data entry separately in terms of reported models. Where models differed within a trust, we asked for detail on this in open-ended data. Written consent was provided on the first page of the survey, which had NHS research ethics committee approval (reference: 23/SW/0028; HRA 316667). Unit name as a mandatory field enabled follow-up of non-responders. The SmartSurvey (V.2024.2.14.10175) was used for data collection. Non-responders were sent several reminders to encourage high response rates. The survey was promoted at national conferences, CC3N Forum meetings and via social media.

### Data management and analysis

**Figure 1:**
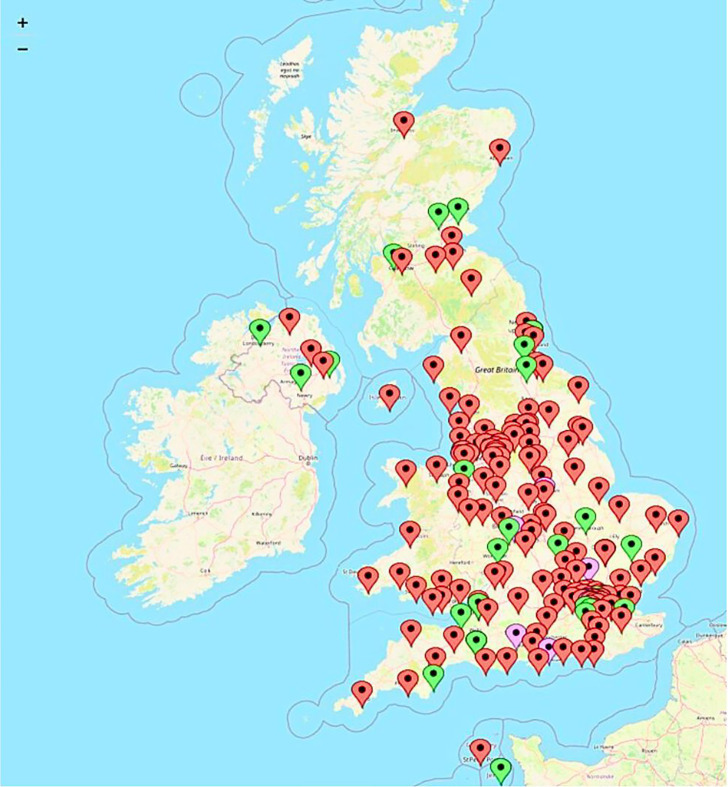
Map of hospital sites from which responses were received.

All data were cleaned and cross-checked by the study team to ensure there were no duplicate submissions or where the survey had already been completed at the trust level. Duplicates were identified and removed prior to analysis. Partial survey responses were assessed for completeness of closed questions (bed number, establishment and nurse to patient ratios) by the study team. Responses were either deemed eligible partial responses and included within the overall response rate or classed as unusable (where no meaningful data regarding staffing were yielded). Data anomalies were double-checked by the chief investigator who contacted site nominees (the designated unit or units senior nurse manager, or person nominated by unit head) to ensure data accuracy. The final survey responses provided strong representation of the UK, Channel Islands and Isle of Man (figure 1). Numerical data were cleaned and exported from Excel into SPSS V.25 and descriptively analysed using proportions, means, medians and SDs. Open-ended data were treated as qualitative, and free-text analysis^17^ was applied to derive coding categories directly from the data (see online supplemental file 1). Data were integrated for presentation of results.

## Results

### Responses

A total of 196 responses were received for 300/331 units (representing 155 trusts and 231 hospitals). 162 of these surveys were retained; nine duplicates were identified and 25 partial responses which did not meet the required minimum data were removed, leading to a total of 162 individual responses from 231 hospitals for analysis and a final response rate of 70.1%. Responding nurse leads often covered several ICUs within their organisation. We report on both unit and trust-level data, where relevant. Open-ended responses provided qualitative explanatory data for some of the survey fields (online supplemental data file 1, table 1).

**Table 1:** Descriptive statistics of unit characteristics

Survey item	Mean (*SD*)	Median	Range (min/max)
Total number of beds per unit (n=162).	26.36 (21.48)	18	5–112
Total number of Level 2 beds per unit (n=147).	10.96 (8.86)	8	0–46
Total number of Level 3 beds per unit (n=159).	15.67 (15.33)	10	2–88
Total number of funded and approved whole-time equivalent registered nurses (RNs) (n=149).	121.20 (97.82)	86.28	18.15–512.29
Total number of funded and approved whole-time equivalent RNs in post (n=142).	116.53 (89.88)	80.52	16.85–492.24
Number of funded and approved whole time equivalent for non-RNs (including Healthcare support workers, registered nursing associates and trainee nurse associates) in post per unit (n=138).	13.68 (12.63)	10	0.8–81
Vacancies (%) (n=138)	7.47 (8.26)	6.07	−5.6* to 39.52
Proportion (%) of RNs with a postregistration qualification (n=140).	46.88 (12.28)	49	10–78
Total ICU nurse (registered and non-registered) staffing requirements per bed (eg, whole time equivalent nurses required per bed) (n=105)**.**	5.52 (0.59)	5.5	3.50–7

* Minus figure is indicative of over-recruitment of staff, permitted temporarily in certain Ttrusts.

ICU, intensive care unit.

Survey nominees (respondents) included the matron (senior/head nurse) of the critical care unit (n=89/162; 54.9%), lead nurses (n=21; 13%), unit managers (n=11; 6.8%), heads of nursing (n=9; 5.6%), senior charge nurses (n=7; 4.3%), nurse consultants (n=6; 3.7%) and senior matrons (n=5; 3.1%). The remaining 14 respondents (8.6%) included deputy directors and directors of nursing, senior nurses, charge nurses and a medical lead.

### ICU unit characteristics

Different sizes and types of the units were reported. Among 300 ICU units, 55.6% (n=90) comprised a single unit, 24.1% (n=39) had two units, 9.9% (n=16) had three units, 4.9% (n=8) had four units, 1.2% (n=2) had five units and 4.3% (n=7) had six units. Unit types were predominantly general ICUs (n=208; 69.3 %), with the remainder being cardiothoracic (n=35; 11.7%), specialist (n=22; 7.3%), surgical (n=17; 5.7%), neurological (n=13; 4.3%) or medical (n=5; 1.7%).

The mean number of critical care beds for each unit within trust sites was 26.36 (21.48; range 5–112); this included funded level 2 beds (mean: 10.96 (SD: 8.86)) and level 3 beds (15.67 (SD: 15.33)) (table 1). Though most units were funded to a maximum number of level 2 and 3 bed numbers, many of these respondents indicated that they used the beds flexibly according to patient acuity and need. Some units were able to flex all of their beds to up to level 3 (full ICU) capacity, exceeding their funded level 3 bed capacity (online supplemental file 1 outlines illustrative quotes). The exception to this was a specialist liver unit which was funded and staffed for level 3 beds, regardless of patient acuity. Bed mix was further managed flexibly across units on different sites, within one NHS Trust, and this was linked to staff being moved across sites (between ICUs on the same hospital site and across the city to ICUs within the same trust) to support patient need and changes in staffing requirements.

One unit noted a continued trend of exceeding their funded Level 3 capacity due to increased patient admissions with increased acuity. One unit reported a greater number of level 0/1 patients due to challenges around bed flow, linked to increased workload for nurses who were required to care for these patients often in addition to higher acuity (level 2 and 3 patients), while another was experiencing an increase in level 2 bed occupancy and longer patient stays because of the removal of the dedicated high dependency unit (providing space solely for level 2 patients).

### Workforce model and characteristics

The calculation for the average nursing requirement based on Whole Time Equivalent (WTE) per bed was 5.52 (SD: 0.59; range 3.5–7), also referred to as ‘establishment’. Establishment is predicated on a nominally ‘fixed’ number of beds (usually around 75% level 3 beds and 25% level 2 beds, reviewed yearly for each unit by commissioners). The average number of funded and approved WTE RNs was 121.20 (SD: 97.82) ranging between very small units to large trusts (range 18.15–512.29) (table 1). Vacancy rates are reported at 7.5% (SD: 8.26), but some were over establishment (had more nurses in their staff establishment than their establishment calculations permitted) and one had a nearly 40% vacancy rate.

Non-registered nursing staff formed an important part of the nursing workforce, with most units employing a headcount of around 13.7 (SD=12.63) staff in assistive roles (Nursing Associates-Registered (NARs), healthcare support workers (HCSWs) and trainee nurse associates). The proportion of RNs who held a postregistration ICU qualification (with reported variation in interpretation drawn from open-ended contextual data) was 48.9% on average (SD: 12.28). Open-ended responses indicated that staffing requirements were calculated predominantly based on the number of level 2 and 3 beds, although some reported the influence of patient dependency or acuity and national guidance (using Guidelines for the Provision of Intensive Care Services V.2 (GPICSv2)).^9^

### Post-COVID-19 establishment change

All except four of the units reported using GPICSv2 guidelines, with only two units not using a 1:1 model (four units did not use the 1:2 model for level 2 patients). These were specialist units, that is, burns unit (with additional staffing guidelines). Two different units (one of which was a cardiothoracic recovery unit) indicated using 1:1 for all patient acuities. One unit reported diluted nurse to patient ratios due to lack of staffing resource during periods of increased activity. 11.3% (n=16) reported decreased staffing costs (associated with vacancy gaps); however, increased staffing costs were reported by 25.5% (n=36/141). A change in ICU nurse staffing establishments since COVID-19 was reported by 44% (n=102) of participants versus 117 (50.4%) stating no change (and 13 (5.6%) stating do not know). Changes included an increase in establishment, linked to an increase in bed base. Open-ended responses, provided by some, indicated an increase in level 1, 3 and 4 (level 3 bed also providing highly specialist care for example, extracorporeal membrane oxygenation) beds, most frequently level 3.

‘Units pre-COVID only had an establishment for 40% of patients requiring 1:1 care, this is now 70%.’ (Site ID 103)

Reported increases in staffing numbers related to an increase in Band 5s (Band 5s are junior nurses, the lowest grade of RN in the UK, comprising the largest portion of the UK workforce—up to Band 8s who are the most senior nurse leaders in ICU), based on high attrition of experienced staff post-COVID-19.

‘We have been allowed an increase of 14 Band 5 WTE since COVID as we are now running more level 3 beds. This increase has not been funded officially so I incur an overspend each month*.*’ (Site ID 15)

‘post-COVID we have had a reduction in Band 6 posts and an increase in Band 5 posts.’ (Site ID 82)

Eight respondents noted an increased number of staff in assistive roles to support with additional level 3 capacity or night shift cover and as a legacy from COVID-19.

In addition to seeking changes in terms of perceived and reported increases/decreases in staffing, we also asked questions on the perceived impact of staffing and allocation changes since COVID-19. 14 respondents (total n=141; 9.9%) reported an increase in RN to patient ratio and eight (5.7%) of the participants reported a decrease in this ratio. The numbers of critical care qualified nurses to patients were reported to have increased for 15 units (10.6%) versus a decrease reported by 26 units (18.4%). NARs were reported to have increased (n=22/141; 15.6%) alongside HCSWs (n=35/141; 24.8%).

**Figure 2:**
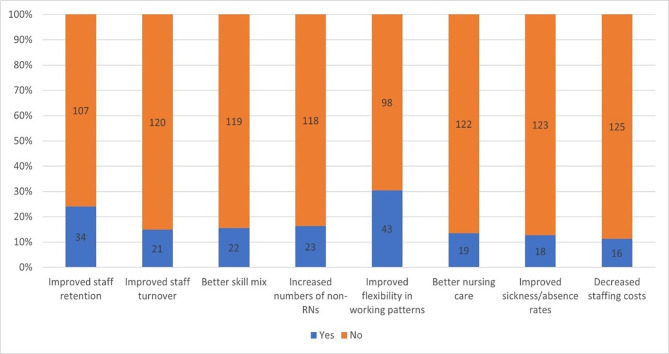
Changes since COVID-19 in critical care nurse staffing. RN, registered nurse.

When asked about reported benefits of current staffing models since the pandemic, 34 participants (total n=141; 24.1%) reported improved staff retention and 21 (14.9%) reported improved rates of staff turnover (the proportion of vacancy each year) after COVID-19 (figure 2). 16 (11.3%) units reported decreased staffing costs (associated with vacancy gaps, see figure 2). Improved flexibility in working pattern was reported by 43 (30.5%) of respondents. 22 units (15.6%) perceived skill mix to be better since COVID-19. Counter to this, when asked about what had worsened, 38 (27%) participants reported worse staff retention and 37 (26.2%) participants reported worse turnover since COVID-19 (figure 2). A fair proportion, 57 units (40.4%), reported skill mix to be worse, and increased staffing costs were reported by 25.5% (n=36/141).

Open-ended data indicated disadvantages to current staffing models in place, including staff attrition and retention, citing promotion opportunities elsewhere, burnout post-COVID-19 and lack of value and recognition of specialist skills.

**Table 2:** Incident and quality data report from ICUs

Survey item (n=141)	Patient safety event		Staff event	
	Frequency (N)	Percentage %	Frequency (N)	Percentage %
Unplanned extubation	117	83	50	35.5
Vasopressor infusions running out	95	67.4	46	32.6
Accidental disconnection of arterial line	90	63.8	43	30.5
Accidental disconnection of central line	101	71.6	45	31.9
Patient falls	131	92.9	53	37.6
Infection rates	125	88.7	56	39.7
Pressure ulcer incidence/prevalence	132	93.6	56	39.7
Medication errors/incidents	125	88.7	56	39.7
Nurse sickness/absence rates	52	36.9	91	64.5
Staff retention/turnover	44	31.2	86	61
None of the above	2	1.4	1	0.7

Participants reported incidents in the context of staffing (table 2) and how data were used to report against the quality of nursing care. The most observed patient safety events were pressure ulcer incidence (n=132/141; 93.6%), followed by falls (n=131; 92.9%) and medication incidents (n=125; 88.7%). Nurse sickness/absence rates and staff retention and turnover were the predominant staff events (n=91/141; 64.5% and n=86; 61%, respectively). The quality of nursing care data was used by nurse leaders to report on patient safety incidents, to monitor staffing compliance and to support workforce review, staffing business cases and for wider learning.

## Discussion

In this survey, we sought to understand current staffing models and the legacy impact of COVID-19, when alternative staffing models were widely in use.^13^

Key findings from this research are a reduction in critical care qualified nurses providing direct care to patients since the COVID-19 pandemic and an increase in non-registered staff being used, alongside worsened turnover and retention. Mechanisms to support staffing vary from flexible working, which has increased, to using national guidance to support safe use of staffing models.

While our survey results demonstrate that national guidelines^9^ and the national service specification^7^ help guide staffing requirements, with almost every unit attempting to adhere to 1:1 and 1:2 ratios, the picture remains unclear as to how staff are allocated at the bedside. The adaptive models described during the pandemic^13 18^ have receded, with units returning to deploying staff to nationally guided ratios. Our data also indicate a worsening in skill mix, within a widespread return to nationally-guided ratios, suggesting that staffing is worse and that staffing must be considered beyond numbers.

In our survey, ratios of RNs to patients appear relatively stable while numbers of critical care qualified nurses to patients have decreased for more units (18.4%) compared with when it increased (10.6%), showing that while for some units ratios had improved, for a greater number it had worsened. The number of non-registered staff has increased (24.8%), suggesting alternative solutions for how to address patient dependency needs and staffing requirements, in the face of ongoing ICU nurse shortages (figure 2).^6^

The most significant benefit of staffing models currently in use was perceived improved flexibility of staffing patterns (n=43, 30.5%) since the COVID-19 pandemic. And while improved staff retention (n=34, 24.1%) was reported by some, a larger number reported worsening turnover, and retention was also reported, indicating a mixed picture, with some form of stabilisation of the workforce in only some units. The high attrition associated with the COVID-19 pandemic may have had a legacy impact, potentially because of high levels of burnout and significant impact on staff well-being and job satisfaction.^19^,^21^ Staff retention and turnover are distinct but related concepts in the literature.^22^ A key difference is that retention can indicate retained employment in the ICU, but possibly in a different position (eg, non-direct care delivery role). With the advent of newer roles, like family-liaison nurse roles in ICU, this might have an effect on how nurse leaders responded to these linked questions.

The increase in staffing expenditure (n=36; 25.5%) could be viewed as both positive and negative; with the increase not necessarily linked to increased ICU RNs, but potentially to increased temporary staff usage based on the high attrition in experienced nurses seen post-COVID-19, as evidenced in the open-ended data. Moreover, nurse leaders reported this as an added pressure. Importantly, nurse team composition was seen to have a clear effect on patient mortality, with an increase in temporary staff leading to higher patient mortality.^23^

Higher critical care nurse staffing was associated with increased family satisfaction,^14^ and staff outcomes included reduced burnout, less intention to leave and increased job satisfaction. International studies undertaken in China and Korea report similar findings associating higher nurse staffing levels with improved patient mortality.^24^,^26^

Most saliently, perceived skill mix was considered to be considerably worse since COVID-19 (40.4%). A recent study in Australia highlighted how lower levels of critical care RNs (CCRNs) were directly linked to mortality. Units with 50–75% CCRNs have higher patient mortality risk than those with more than 75% CCRNs (adjusted OR 1.21 (95% CI 1.02 to 1.45)). The biggest barrier and risk factor for safe, effective care in pandemic-affected ICUs across the UK was the lack of ICU-qualified nurses,^15^ also noted in a Swedish study of missed care during the pandemic.^27^

These results raise questions pertaining to the role of non-registered staff in ICU and how much direct care they provide. We noted a relatively small vacancy gap; however, skill mix was cited as problematic, in keeping with earlier findings from the preliminary SEISMIC study.^15^ An increase in staffing costs and poorer skill mix was reported in the open-ended data as related to international recruitment drives and relocation of nurses at a higher volume but lower banding and associated training and funding needs.

Building on previous studies, there is a pressing need to reconsider acuity, determined solely through organ failure,^8^ as the key determinant of nurse-patient ratios. Using acuity in this way risks undermining nursing workload,^28^ and using patient-based workload like acuity poorly correlated with perceived ICU workload.^29^ Nursing dependency is managed through professional judgement used alongside nationally guided ratios,^30^ as evidenced in this study. Research in NICU has also found that subjective views of workload are associated with missed care,^31^ demonstrating the importance of workload perceptions for patient outcomes. Issues such as technical competency and proficiency, experience, banding or grading of ICU staff, the promotion of staff well-being, direct care provision and mentorship and supervision of others are examples of additional factors influencing nursing capacity.

In terms of the effect of staffing and while nurses equate safe staffing to patient safety,^15^ our study highlights that patient and staff incident reporting have distinct indicators and staff event reporting in correlation to clinical incidents is low (vasopressor infusions running out, patient falls). Monitoring of adverse events for quality of nursing care by nursing leadership, requiring a trust-level response, dropped across all survey items. This corroborates with findings from Falk *et al*,^27^ who outlined that during the pandemic, nurse/patient ratios were breached consistently, leading to missed basic nursing care. In our study, there was an increase in sites not formally monitoring any adverse events for quality of nursing care to senior level. This infers that leadership responsibility in linking and escalating patient and staffing concerns is lacking in many hospitals, especially at times of crisis. Wynne *et al* suggested reform around pandemic staffing needs to be nurse-led,^32^ which we would support. However, pinpointing the relationship between staffing and clinical outcomes has historically been challenging,^14^ in part due to the inconsistency with which outcomes are measured or reported, as we have shown in our survey.

### Strengths

A major strength of the study was its high unit-level coverage of 90.0% of all UK units and known denominators, signalling engagement on the topic and need for ICU staffing challenges to be addressed. This provided a comprehensive picture of nurse staffing models in the UK, and our open-ended data were important for helping to understand the variation and impact the pandemic had on these models. We have also identified that nearly all of the UK has resumed staffing critical care units according to national guidance recommendations of 1:1 for the sickest patients. Key areas of concern for future research are also highlighted.

### Limitations

Despite piloting, there were ambiguities identified in the variability in data responses, particularly to a question asking how staffing requirements are calculated, which would have benefitted from greater clarity around whether this included non-registered staff. When checking outlying data with respondents, we determined there was variability in terms of how this was interpreted, accounting for the heterogeneity in responses, so we cannot be sure if sites have reported this as solely for registered nursing staff and limiting interpretation in this response. Survey results do also not account for the normalised practice of ICU nurses being released to other understaffed ward areas.^33^ Identification of sites and suitable respondents was time-consuming due to the number of trusts, site and unit organisation, especially in larger trusts. Identification of nominees created some overlap and duplication of data, which was addressed at analysis through site identifiers. A national registry of trusts, sites, units and corresponding staffing and bed capacity would support readiness for research and promote unit connectivity.

Ambiguity still arose in the present survey, for example, 50.4% of respondents answered ‘no’ to seeing a change in ICU nurse staffing establishments since COVID-19 (Q9a). Corresponding free text answers then divulged issues surrounding skill mix, patient safety, staff well-being and retention. Results also revealed anomalies in perceived advantages and disadvantages in the way critical care nurses are allocated (Q10). Results do not account for variation in the grades of ICU nurses, 51% of whom did not hold an ICU qualification. Defining the terminology of ‘ICU nurse’ would clarify what constitutes a nurse working in ICU and those with ICU qualifications.^28^ Inconsistent approaches to measurement were highlighted as a barrier in providing recommendations for safe staffing.^14^ A better understanding and ability to adapt staff modelling, as highlighted during the COVID-19 pandemic, could support nurse leaders to interpret and analyse in real-time and be responsive and adaptable ‘on the ground’. We relied on staff to report differences in models and also describe in more detail in open-ended data; there could have been further variation in practices not reported beyond the simplistic adherence to guidance. The bidirectionality of survey questions also meant some inconsistency in answers.

We were unable to determine, through survey methods, the ability of nurse leads as local experts to influence staffing models, although nearly all used national guidance,^9^ suggesting GPICSv2 remained a useful framework for organising staffing. Through qualitative data (interview and ethnographies), alongside large-scale operational modelling examining patient outcomes and staffing roster data, the wider SEISMIC-R study (ref: NIHR 135168) seeks to investigate the relationship between staffing and clinical outcomes.

## Conclusion

This survey study adds to the limited existing literature outlining the current unit and workforce characteristics of ICU units across a wide geographical spread and unit types, and how these have changed since COVID-19, particularly in relation to poorer skill mix, despite ratios returning to nationally guided numbers. Acuity models are still used to determine nursing ratios and underpin national guidance, which risks undermining nursing, care delivery and outcomes like care quality and retention, especially when dependency is high and unaccounted for in numerical staffing plans. While most units aimed for a staffing model compliant with national staffing guidance, there was a suggested legacy impact of the increased use of non-registered staff during COVID-19, indicative of continued alternative models in use in some units. The impact of how nurse staffing data is used to support and contextualise incident data reporting is also evident from our research. More research is needed to clarify how staffing groups (RNs vs non-RNs and within RNs) are being used to allow for increased flexibility in working patterns, fill the vacancy gaps and deliver clinical care interventions.

## Supplementary material

10.1136/bmjopen-2024-088233 online supplemental file 1

10.1136/bmjopen-2024-088233 online supplemental file 2

## Data Availability

Data are available upon reasonable request.
