# Supplementary material for: National survey evaluating the introduction of new and alternative staffing models in intensive care (SEISMIC-R) in the UK
Source: BMJ Open. 2025 Apr 10;15(4):e088233. doi: 10.1136/bmjopen-2024-088233 (PMC11987101; doi:10.1136/bmjopen-2024-088233)
Supplement: online supplemental file 1 [file bmjopen-15-4-s001.docx]

**Supplemental file 1: Qualitative data tables**

Table 1: Summary of themes and sub-themes, frequency of occurrence and illustrative quotes.

| **Survey question** | **Theme** | **Total frequency of theme (n)** | | | **Illustrative quote** |
| --- | --- | --- | --- | --- | --- |
| **Q2. How many beds does each unit have?** | | | | | |
| 1. Unit description | | | | | |
| Total number of funded beds  Physical vs funded beds  Surge capacity | | | 39  15  5 | | - “We are currently funded for 17 level 3 equivalents at the [site] and 15 at the [other] site” [SID 109] - “Our unit is funded for 12 general level 3 beds, 6 general level 2 beds and 2 level 3 speciality burn beds.” [SID 131] - “Unit 1; ITU funded for 7 L3 and 4 L2 however we only have 10 beds so if we needed to accommodate full complement we would need to use L2 beds in unit 2….” [SID 114] - “There are a total of 28 physical beds across 2 floors. We are currently running 22 beds due to funding.” [SID 115] - “We have been reduced back to the original 10 beds [from 12 beds] and use an escalation bed in the Theatre Recovery if we need to.” [Site ID 118] - “Short term surge +2 3 when required. Manage surge capacity by curtailing elective surgical HDU cases during times of peak demand.” [Site ID 77] |
| Flexing level 2 and 3 bed mix to meet patient need | | | 89 | | - “Our beds are flexed to suit patient dependency and acuity - we have staff on shift to accommodate [a maximum of] 5 level 3 patients or equivalent.” [SID 111] - “Bed base will flex up on L2 and Down on L3 or vice versa when required.” [SID 4] - “Except for our specialist Liver unit (15 level 2 + 4 level 2) all of our other units are capable of flexing up to 100% L3 capacity if required (a bed is a bed).” [SID 95] |
| Actual bed mix | | | 16 | | - “Unit 1, 4 & 6 fluctuates regularly and can be over Level 3 capacity.” [SID 122] - “Fluctuation varies throughout the year with an increase up in level 3 activity during winter months.” [SID 90] - “…44 beds and currently funded for a 50/50 split of L2 and L3 beds. However in the past 18months our acuity has increased and we are more frequently caring for in excess of 22 L3 patients. On occasion this had peaked to 35 L3s.” [SID 16] |
| Case mix | | | 8 | | - “Most of the admissions are elective (planned surgical admissions) and also medical non elective admissions (haematology/Oncology related emergency admissions)- 70% elective and 30% non-elective” [SID 59] - “6 level 2 beds, mainly elective orthopaedic admissions. However unplanned admissions with deterioration from Spinal Injuries Unit, surgical wards and medical/rehab ward.” [SID 72] - “The Cardiac ICU case mix is approximately 25% medical, 15% emergency surgical and around 60% planned urgent surgical admissions.” [Unit 1, SID 60] |
| Unit geography | | | 8 | | - “ITU- 9 physical single rooms; establishment ICS dep of 8 - 8 bedside nurses plus 1 shift leader. 8 level 3 beds” [SID 123] - “We also have a 17th bed which is an isolated side-room which is in the footprint. If we use this bed we may need to close another bed in the main unit.” [SID 6] - “All individuals rooms so ability to look after level 2 & 3 patients.” [SID 30] |
| Management of unit | | | 1 | | - “Unit 2: Medical HDU …I do not manage this unit. It is manged by Acute medicine and covered by Acute Medical Physicians. There is collaboration working with the ICU Consultants….[unit 1 and 3] are managed by ICU Consultants 24/7…” [SID 13] |
| 1. Strategies to manage bed mix fluctuations and delivery of care at times of increased capacity and acuity | | | | | |
| Flexible staff utilisation across units and sites | | | 5 | | - “We have two physical units, one at each hospital…, but we use them as one unit, so staff move freely to where patient need is.” [SID 107] - “…We are funded for a dependency of 20 nurses across the service, the nurses work very flexible across the two services.” [SID 7] - “All staff work between both the adult and paediatric unit.” [SID 49] |
| Increased patient to staff ratios | | | 1 | | - “…at times of peak activity - staffing ratios for all patients, where they cannot be mitigated by alternative resource strategies, are flexed to maximise safety for all patients and to deliver care for all - this means that sometimes, ratios of patients at any level of acuity may be higher than our goal.” [SID 110] |
| Moving patients | | | 1 | | - “We also move patients internally to support safe staffing.” [SID 122] |
| Use of assistant practitioners | | | 1 | | - “We also have a cohort of Assistant Practitioners so can increase L3 capacity using a Registered Nurse/AP pairing” [SID 22] |
| **Q8. How the total ICU nurse staffing (registered and non-registered) is calculated** | | | | | |
| 1. Based on level 2 and 3 bed numbers | | | | | |
|  | | | | 30 | - “Based on 18 level 3 and 12 level 2…” [SID 8] - “Registered staff are calculated as that required for 6 x L3 and 5 x l2 beds…” [SID 147] - “…Staffing model built on 4 L3 beds, 4 L2/1 beds…” [SID 77] |
| 1. Based on dependency | | | | | |
|  | | | | 5 | - “33 RN's to cover dependency of 33, plus 5 coordinators, therefore 38.” [SID 78] - “We have 6 / 7 beds in ICU and are funded for a dependency of 5. We take a mix of level 2 & 3 patients up to a dependency of 5 or 7 patients as a maximum.” [SID 120] |
| 1. Based on acuity | | | | | |
|  | | | | 3 | - “We are established for an acuity of 5 in critical care which can be a mix of L3's and L2's to the physical bed base of 8” [SID 125] - “Not based on beds based on elective activity and acuity” [SID 48] - “Based on acuity” [SID 34] |
| 1. Calculated uplift | | | | | |
|  | | | | 3 | - “20% uplift” [SID 6] - “uplift is not the same as this [safe staffing] tool.” [SID 19] - “+ 21%” [SID 62] |
| 1. Other | | | | | |
|  | | | | 1 | - “14WTE per day” [SID 45] |
| 1. Using staffing guidelines and tools | | | | | |
| GPICS | | | | 6 | - “plus additional supervisory nurse in charge for every 10 beds, according to GPICS” [SID 86] - **“**Registered staff are calculated as that required for 6 x L3 and 5 x l2 beds in accordance with GPICS” [SID 147] |
| Other | | | | 3 | - “rcn [Royal College of Nursing] guidance used of 7 WTE per bed.” [SID 5] - [inserted link to nursing times article about using the safe staffing tool] [SID 19] |
| **Q9b. Have ICU nurse staffing establishments changed since Covid?** | | | | | |
| 1. Changes to bed base | | | | | |
| Increase in absolute bed numbers  Increase in level 1 beds  Increase in level 2 beds  Increase in level 3 beds  Increase in level 4 beds  Unfunded increase (overspend) | | | | 19  1  4  6  1  2 | - “We extended our bed base from 20 to 28 beds in 2022, utilising a separate ward for the additional 8 beds” [SID 79] - “uplift in nursing establish for both units inline with increase of bed base. Have gone from 12 beds in unit 1 to 16 and 13 beds in unit 2 to 16 over the last 3 years” [SID 1] - “Increased from 15 beds to 19 beds, and now recruiting into and opening the bed base over the next 12 months.” [SID 71] - “4 L1 beds were attached to a ward area. Level 1 beds increased to total 12, increase by 8.” [SID 114] - “opened HDU 8 level 2 beds” [SID 123] - “additional 5 HDU funded beds added to the nursing establishment” [SID 27] - “additional funded 3 level beds” [SID 38] - “Total number of Level 3 beds has gone from 32 to 36 across both units since COVID-19” [SID 54] - “Unit 3- expansion of beds for level 4 so establishment increased.” [SID 39] - “We have been allowed an increase of 14 Band 5 WTE since COVID as we are now running more level 3 beds . This increase has not been funded officially so I incur an overspend each month.” [SID 15] - “Unit 1 opened 2 unfunded beds in response to pandemic. Unit 3 opened 2 unfunded beds in response to pandemic. Increase in L3 beds lead to need for supernumerary nurse as 10 increased to 12 beds.” [SID 24] |
| Reduced number of beds | | | | 4 | - “We reduced bed numbers to ensure patient/staff ratios were good and side room and the different geographical areas were staffed properly” [SID 17] - “Reduction of bed base from 23 beds to 18 beds from May 2023” [SID 10] |
| No change in bed base | | | | 2 | - “Same number of open beds following covid on both units…” [SID 32] - “There has been an increase in nursing establishment but not an increase in the physical beds available (escalation plan notwithstanding)” [SID 81] |
| 1. Actual or planned increase in establishment | | | | | |
| Registered staff  Band 5  Band 6  Band 7 | | | | 30  3  6  4 | - “uplift in nursing establish for both units inline with increase of bed base.” [SID 1] - “Increase in budgeted establishment of 5 WTE RNs” [SID 65] - “…recruited more band 5s than establishment to ensure sufficient qualified nurses” [SID 25] - “post covid…increase in Band 5 posts” [SID 82] - “We have been allowed an increase of 14 Band 5 WTE since COVID as we are now running more level 3 beds” [SID 15] - “…now have Band 6 Clinical educator and 0.4 wte for Follow up Clinic.” [SID 107] - “We increased our Band 6 RNs in response to covid as it was nationally recognised there was a lack of senior support during covid.” [SID 9] - “Band 6 from 21.47 TO 25.76 WTE” [SID 6] - “…secured improvement to band 7 numbers from 2 to 3.6 to achieve 7 day cover to achieve clinical operational decision making 7 days a week.” [SID 117] - “Increase in Band 7 numbers to support 7 day cover [on unit 1 and 2]” [SID 24] |
| Training and utilisation of assistive staff | | | | 12 | - “To support we have recruited more HCA's [Healthcare Assistants] then budgeted for.” [SID 13] - “We now have RNA's [Nursing Associates - Registered] (looking after 1 level 2)” [SID 89] - “Unit 2 had just started AP [Assistant Practitioners] programme and now has qualified AP group alongside.” [SID 24] |
| Medical staff | | | | 1 | - “Increased nursing and medical establishment to keep all 12 beds open following the covid funding.” [SID 19] |
| Addition of supernumerary staff to support unit geography | | | | 6 | - “ITU requirement was increased to support….increase [in] supernumerary runners from 3 to 4 to support 10 side rooms” [SID 129] - “Following Critical Care network Peer review, we increased our Band 6 establishment to allow for a second supernumerary co-ordinator as per GPICS for over 10 patients.” [SID 30] |
| 1. Actual or planned decrease in establishment | | | | | |
|  | | | | 7 | - “Not due to the pandemic due to altered shift patterns reducing the overall required establishment” [SID 126] - “We are still working with an unapproved shift plan. We are working with a shift plan that was created in 2015 and over budget. However, I have been told to reduce the staff” [SID 76] |
| 1. No change in establishment | | | | | |
|  | | | | 5 | - “Same number of open beds following covid on both units, so no change to staffing.” [SID 32] - “GPICS Compliant pre/post 2020. Nothing has changed” [SID 95] |
| 1. Addressing gaps in staffing on shifts | | | | | |
|  | | | | 6 | - “…to achieve 7 day cover to achieve clinical operational decision making 7 days a week. There is a band 7 on duty 7 days a week.” [SID 117] - “HCA x 1 for night shift added to WFM (previously had no HCA on nights)” [SID 107] |
| 1. Recruiting to fill vacancies post-Covid | | | | | |
|  | | | | 11 | - “We had 50% staff leave or reduce their hours post COVID and it has been hard to recruit…” [SID 87] - “We have gaps at Band 6 level-difficult to recruit experienced B6s.” [SID 35] - “Post pandemic we have a depletion of some senior staff however we have had a positive recruitment drive for both band 5 and band 6 staff.” [SID 6] |
| Q10) How has the way critical care nurses are allocated changed since the pandemic | | | | | |
| 1. No change in nurse staffing allocation | | | | | |
|  | | | | 71 | - “Our allocation has remained the same.” [SID 12] - “It has remained the same.” [SID 58] |
| 1. Increase in Registered Nurses to meet increased ICU demand | | | | | |
|  | | | | 5 | - “The number of registered nurses has increased in line with increased L3 activity and L2 patients requiring 1:1 nursing care.” [SID 103] |
| 1. Actual and considered changes to the number of non-registered staff | | | | | |
| As a solution to recruitment challenges  Due to unit geography | | | | 8  1  3 | - “We have also had TNA's [Training Nursing Associates] qualify since the pandemic which has increased the number of RNA's [Nursing Associates-Registered].” [SID 104] - “Decreased numbers of HCA [Healthcare Assistants] due to recruitment issues.” [SID 36] - “Healthcare support workers increased during covid for support and PPE for staff. These staff are now back to one HCA in each of our 3 pods.” [SID 35] - “…recruitment has been very difficult and the thought processes are the utilisation of RNA in ICU, however this has significant difficulties in a smaller unit such as ours in regards to leading on care and IV medications and management, especially if our nurse in charge is not supernumerary.” [SID 74] - “We have factored the supernumerary clinical support required to safely care for patients in the side rooms.” [SID 51] - “Increased due to bed base expansion and geography of unit including increase in isolation rooms” [SID 88] |
| 1. Decreased Registered Nurse to patient ratios during and after Covid | | | | | |
| Legacy of Covid  Increased safety concerns and incidents | | | | 6  2  1 | - “Durning the pandemic nurse:patient ratios were stretched due to the volume of patients admitted to critical care. We have now gone back to GPICS staffing ratios.” [SID 104] - “Staffing ratio's have had to be flexible during waves of covid and education/admin time has been lost to clinical time for safety.” [SID 102] - “We occasionally nurse long term ventilated weening patients a 1:2 inline GPIC guidelines which we did not do as much before covid.” [SID 109] - “… during the pandemic nurse patient ratios increased and this has become an accepted norm within the trust we manage our own staffing and report into the staffing cell if we have exhausted options and our staffing is red there is little support available and the expectation is to increase nurse patient ratios (become normalised).” [SID 127] - “At times we have nursed 1:2 for level 3 patients, sometimes utilising unregistered staff. We have seen an increase in DATIX incidents and safety concerns with this.” [SID 122] |
| 1. Recruitment of international nurses to address vacancy gaps and meet establishment requirements | | | | | |
|  | | | | 4 | - “We have had a large increase in international nurses to fill our vacancies.” [SID 61] - “Due to an enforced expansion, we had to take on over 100 nurses requiring novice training, many of whom were internationally educated and recruited.” [SID 64] |
| 1. Fewer critical care qualified nurses to patients | | | | | |
| Reduced interest from staff in completing ICU course | | | | 4  2 | - “Lost a few bend 6s to career progression and a move out of ICU post pandemic.” [SID 83] - “Several experience staff nurses have retired or left for alternative roles.” [SID 144] - “Our % of Critical Care qualified staff dropped post covid as experienced nurses left and staff were less inclined to complete a university course.” [SID 49] - “Less people want to do the critical care course” [SID 83] |
| 1. Other | | | | | |
| Flex elective activity according to available beds | | | | 1 | - “… cardiac level 3 unit: Almost all patients are elective and as such activity can be flexed according to available beds and staffing by adding/cancelling lists...” [SID 110] |
| Flex staff on and off to meet unit demands | | | | 1 | - “…maintain nurse:patient ratios by using flexible working with our own staff as unable to get help from the wider organisation with skilled CCN's and unable to get agency.” [SID 111] |
| Increased allocation of supernumerary nurses to patients to reduce staffing costs | | | | 1 | - “More likely to use the Nurse in Charge or take education nurses into numbers rather than go out to Agency.” [SID 119] |
| Staff deployment to other ICU units and wards | | | | 3 | - “staff are now more frequently asked to redeploy from ITU to other areas” [SID 130] - “Increased staff deployment across critical care (multiple units on one site) to cover shortfall of staff in other units and to reduce bank & agency spend…” [SID 60] |
| Increased numbers of critical care qualified nurses to facilitate internal recruitment | | | | 1 | - “We are putting more people on ICU course to internally recruit...” [SID 25] |
| Nurses increasingly allocated wardable patients due to system pressures | | | | 2 | - “Due to delayed discharges in ICU, one nurse is caring for 3 to 4 ward ready patients.” [SID 146] - “We are faced with more level 1/0 patients on the unit. Therefore the nurse is expected to look after more patients. There is limited bed flow.” [SID 48] |
| Q11a) Have there been any benefits (since 2020) of the current staffing model you are working in? | | | | | |
| 1. Increased establishment | | | | | |
| Assistive roles to support non-nursing tasks and to fill vacancies | | | | 4  8 | - “Greater number of nurses available to cover shifts to GPICS standards.” [SID 85] - “practise educator introduced (included in nursing template)…” [SID 102] - “More HCA's [Healthcare Assistants] has helped with non-nursing tasks workload.” [SID 33] - “Increase in number of RNA's [Nursing Associates-Registered] within unit have greatly supported the workforce.” [SID 140] |
| 1. Improved staff wellbeing and resilience | | | | | |
| Educational and psychological support mechanisms  Improved flexibility in work patterns | | | | 3  8  7 | - “… more resilience in available skilled workforce.” [SID 20] - “There seems to be an improved culture change, sickness has improved since the pandemic.” [SID 109] - “Just starting to see improvements in retention and turnover. We have a psychologist since Covid to support staff.” [SID 13] - “We have implemented Professional Nurse Advocates to assist with staff resilience and education.” [SID 87] - “Flexing on and off supports staff retention.” [SID 45] - “Flexible working has improved our sickness absence rates during reduced acuity as staff are able to take a shift off and work it back when acuity increases.” [SID 111] |
| 1. Reduced staff redeployment | | | | | |
|  | | | | 1 | - “Rarely move staff to the ward due to national guidance document” [SID 83] |
| 1. Reduced staffing costs | | | | | |
| Improved retention and reduced agency use  Due to being under-establishment | | | | 6  1 | - “With recent recruitment we have been able to reduce agency costs.” [SID 146] - “This has… decreased sickness and finance to some degree.” [SID 35] - “Decreased staffing costs due to being constantly under establishment.” [SID 113] |
| 1. Impact of changes made to staffing model unclear | | | | | |
|  | | | | 3 | - “Self rostering has been introduced which may have benefits; still to be evaluated.” [SID 25] - “It is too early to see full results of these ….” [SID 40] |
| Q11b) Have there been any disadvantages (since 2020) of the current staffing model you are working in? | | | | | |
| 1. Establishment calculation | | | | | |
|  | | | | 3 | - “Headroom (23%) insufficient to deliver required education, increased absence and Maternity Leave. (Actual HR >30%)” [SID 53] - “NB trust headroom % exceeded due to sickness absence, maternity leave, other leave, and study leave.” [SID 60] |
| 1. Higher staffing costs | | | | | |
| Due to increased agency and bank use  Due to increased establishment | | | | 7  2 | - “Increased staffing costs related to increased bank and agency rates and usage to cover vacancies, sickness absence, maternity leave, other leave, study leave and supernumerary time for new starters.” [SID 60] - “We have additional runners the numbers that are not funded impacting on staffing costs.” [SID 22] - “Increased staffing costs related to an increased requirement of mental health RN [Registered Nurses] or HCSW [Healthcare Support Worker].” [SID 60] |
| 1. Quality of nursing care post-Covid | | | | | |
|  | | | | 5 | - “Less quality nursing care, again due to that junior skill mix and not having that experience and all other staff around you struggling to support as all as skilled as each other.” [SID 4] - “…quality of care was reduced and it has been difficult to instill 'normal ways' of working into new critical care staff who started with Covid (POD ) Nursing.” [SID 145] |
| 1. Worse staff retention, turnover and sickness | | | | | |
| Staff wellbeing and the impact of Covid  Career progression opportunities elsewhere  Reduction in registered and non-registered staff  Increased agency use to cover gaps in nursing establishment | | | | 12  7  6  4 | - “Reduction in teamworking affected by the above, health and well being and burnt out (no time to recover from pandemic).” [SID 53] - “Band 6 senior staff nurses were affected the most. The responsibility to support the junior workforce and additional staff relentlessly throughout 2020 especially took its toll on this middle band of staff.” - “Seen an increase in staff leaving to seek additional rewards elsewhere, ie higher bands.” [SID 32] - “Experienced staff leave for better work life balance and opportunities to progress and get paid for the skills they have.” [SID 57] - “We have found it difficult to recruit and retain HCSW as their role is very different to that of a HCSW on the wards, which is where many of them move from.” [SID 12] - “We have a band 6 gap of around 30 WTE, which is a 51% vacancy rate at band 6.” [SID 64] - “More agency use to cover shortfall in nursing gaps.” [SID 74] - “… heightened sickness that has required back fill of agency, prior to the pandemic own staff were more prepared to bank and complete overtime…” [SID 127] |
| 1. Disadvantages not perceived as related to staffing model | | | | | |
|  | | | | 12 | - “No, but most of our changes are due to moving into a new hospital in 2022 as opposed to a change post pandemic.” [SID 9] - “…it is rather a result of the changing health care setting not the staffing model that has caused disadvantages.” [SID 40] |
| 1. Worse skill mix due to loss of experienced nurses post-pandemic and increase in junior workforce | | | | | |
|  | | | | 26 | - “Last year we saw 10% of our staff leave for other posts, therefore we now have a very novice workforce. Skill mix continues to be a challenge.” [SID 115] - “Post covid we have generally found a reduction of skill mix -lots of new nurses and experienced nurses leaving critical care…” [SID 49] - “Post Covid the unit lost a lot of experienced staff due to early retirement etc…January 2023 48% of our staff were under 12 months.” [SID 145] |
| Q12b) Is any of the following data used to formally monitor quality of nursing care by nursing leadership | | | | | |
| 1. Data reporting | | | | | |
| Incident reporting and evaluation  Audits  Impact of staffing breaches | | | | 31  8  3 | - “All patient safety incidents are datix then each month datix themes are publicised to the unit…” [SID 127] - “All above incidents would be entered into QSAFE…” [SID 137] - “Patient falls- audits. Infection rates- local audits.” [SID 92] - “Audits completed regularly and shared.” [SID 21] - “…we have seen peaks in all patient safety related incidents when unable to maintain adequate staffing levels.” [SID 134] - “At unit level we collect/compile data which shows consequences of staffing breaches etc…” [SID 114] |
| 1. Organisational oversight | | | | | |
| Governance meetings  Staffing compliance  Care quality and performance | | | | 27  15  4 | - “Discussed during governance safety meetings and governance risk assurance groups.” [SID 9] - “Any issues are discussed monthly at operational governance meeting, which incorporates patient safety.” [SID 23] - “Monthly 1:1 between unit manager and matron review all KPIs reviewing staff retention, staff training, any complaints compliments and any areas of interest.” [SID 40] - “We have performance review meetings where nursing absence and turnover rates are discussed.” [SID 109] - “Falls, infection rates, pressure ulcers and medication errors are all reported, discussed and actioned at weekly clinical indicator meeting.” [SID 75] |
| 1. Learning, training and development | | | | | |
|  | | | | 37 | - “We learn from incidents and put further training on if themes are seen.” [SID 107] - “We have a critical care bi-monthly newsletter and a monthly business matters information update.” [SID 22] - “Trust has just launched PSIF and emphasis is now placed on what learning can be achieved from events.” [SID 29] |
| 1. Finance, commissioning and budgeting | | | | | |
|  | | | | 4 | - “reported and reviewed at CIA [Comprehensive Investment Appraisal].” [SID 141] - “Some events are used as background support for business case to increase nurse staffing or when reviewing recruitment and retention.” [SID 121] |
| 1. Safety mechanisms | | | | | |
|  | | | | 22 | - “There are separate patient safety groups that discusses falls, pressure areas…” [SID 23] - “Three times a week Harms meetings to discuss incidents that may have potentially or actually caused harm to the patients…” [SID 112] |
| Q14) Are there any changes you would like to see in how your staffing in your unit is managed? | | | | | |
| 1. Changes to staffing model | | | | | |
| Staffing calculations and proportion of staff at different grades  Increase in and strategies to support skill mix  Use of Nursing Associates – Registered  Allocation based on unit acuity  Increase in establishment  Allied health professionals  Funded education staff  Assistive roles and support staff  Registered nursing staff  Compliance with national guidelines and service specifications  Supernumerary nurse to support unit geography | | | | 19  12  3  2  3  9  12  6  10  5 | - “…headroom increase to accommodate maternity leave. We have 5 nurses on maternity and this is not covered by the headroom which impacts on the daily staffing levels and critical care capacity.” [SID 146] - “I would like to see how we calculate our staffing per bed (5.5 wte) to be benchmarked with other Trust to ensure equity.” [SID 62] - “More flexibility in moving budget to change skill mix in establishment.” [SID 15] - “Adjustments already made to rotas to ensure even spread of skill mix.” [SID 85] - “… would like to manage in teams i.e. blue/yellow/red. Each team would have a complete skillset…Would like to be more structured in terms of skillset on shift and being able to accommodate certain types of admissions.” [SID 139] - “RNA's [Nursing Associates – Registered] function better in the ward environment, [Nursing Associates – Registered] currently can look after 1:1 level 2 due to limited knowledge and emergency responses.” [SID 89] - “I would think the introduction of Nursing associates in the unit could help with retention of healthcare support workers (those not interested in pursuing nursing career) as they could upskill within their scope of practice.” [SID 59] - “Each unit sees the acuity of other units to understand the allocation more clearly.” [SID 25] - “Might need to increase nursing ratio's on NICU due to acuity of patients they admit.” [SID 27] - “Therapies however is under resourced and would be the next step for staffing investment.” [SID 103] - “Nurses are providing additional support to Physiotherapists/Occupational Therapists /Dietician due to their provision is not to GPICSs standard.” [SID 53] - “I would like to increase my practice based educators team to help support my junior skilled team.” [SID 35] - “I would like the staff to be supported more with additional clinical nurse educators.” [SID 76] - “I would like more CNE provision, currently still at the 1:75 ratio.” [SID 109] - “We would like an increase in non nursing support (ie HCSWs [Healthcare support workers]) to work alongside nursing staff, making a 1:2 ratio easier to manage at times.” [SID 65] - “Need increase in Hca [healthcare assistants] and support workers…Housekeepers and domestics for higher IPC [infection prevention and control] requirement.” [SID 5] - “I would like an uplift in establishment -particularly band 6 /7” [SID 49] - “I would like to see the introduction of ACCPs [Advanced critical care practitioners]” [SID 130] - “Increase in Band 7 senior nurse establishment…” [SID 17] - “Maintaining our registered nurse to patient ratio for critical care (1:1 ICU, 1:2 HDU)” [SID 122] - “To Meet GPIC standards with regard to additional supernumerary nurse for units greater than 10 beds.” [SID 37] - “Increased establishment to take into consideration the use of side rooms, that need support with a 'runner' on the outside.” [SID 68] - “Additional supernumerary RN per shift not providing direct pt care due to geographical layout of unit and number of side rooms.” [SID 60] |
| 1. Increased autonomy for senior ICU staff in making staffing decisions | | | | | |
|  | | | | 5 | - “We would like to employ and interview our own HCAs- this is done centrally and has a high failure rate.” [SID 132] - “The ICU leadership team should be regarded as experts in the speciality field in terms of identify staffing risks and mitigation, allocations and dependency.” [SID 74] |
| 1. Recruitment and retention strategies | | | | | |
| Development and succession planning  Increased flexibility in working patterns  Educational support  Protected supernumerary time and reduced staff redeployment  Increase in funding for and access to training | | | | 27  4  21  10 | - “I would like some more development pathways from band 6 to 7…Financial incentive possibly-band 6 when step 1 achieved, band 7 when 2 & 3 and critical care course.” [SID 121] - “More opportunity to work at a senior level. More role differentiation to aid retention and prevent burnout.” [SID 14] - “I would like to see an increase in band 6 positions to reflect and reward the experience and expertise of the senior nurses currently known as senior 5…” [SID 61] - “…to have an on-call system that staff are paid for even if they aren’t called in.” [SID 139] - “…flexible working thus unit quiet hence staff take time out, unit busy staff work the hours owed thus have a cost saving.” [SID 87] - “Reduction in staff moves to other areas when acuity is low., this would result in improved education and training, staff retention and well being and morale.” [SID 126] - “Supernumerary coordinators should not be included in general staffing numbers when the unit is busy, as they are there to support staff and bank/agency nurses...” [SID 78] - “Increase numbers of staff who can access HDU [high dependency unit]/ICU courses.” [SID 50] - “Better opportunities for staff particularly senior staff that don't necessarily want to be managers bit develop clinical skills eg ACP's [Advanced clinical practitioners]…” [SID 70] |
| 1. Management of patient flow | | | | | |
|  | | | |  | - “Delayed discharged are becoming increasingly problematic with pressures of flow affecting everywhere.” [SID 126] - “Delayed discharges are a regional if not national issue. Discharges to be prioritised at hospital bed meetings.” [SID 98] |
| Q15a) Are you always able to deliver the planned staffing model and what challenges are there to it? | | | | | |
| 1. Sickness and unplanned absence | | | | | |
|  | | | | 53 | - “No always due to on the day sickness.” [SID 4] - “Short term sickness and episodes of emergency leave which cannot be predicted or planned for.” [SID 15] - “Daily challenges with unplanned leave and sickness absence.” [SID 42] |
| 1. Financial constraints on spending on staffing establishment | | | | | |
|  | | | | 6 | “Trust financial drivers to reduce bank and agency spend.” [SID 60]  “Challenges are securing the funding to meet the Gpix guidelines.” [SID 146] |
| 1. Reliance on temporary or non-registered staff to support care delivery | | | | | |
|  | | | | 26 | - “Mostly but heavily reliant on temporary staffing (bank and agency).” [SID 18] - “Not always if our workload increases to more patients than we are funded for, then we are reliant on Agency or using HCA's [healthcare assistants] to support registered staff with level 2 patients.” [SID 26] - “…bank or agency used to maintain unit safety . Rarely need to use agency.” [SID 61] |
| 1. Skill mix | | | | | |
|  | | | | 13 | - “Not always - due to unavailability of appropriately trained nurses.” [SID 119] - “Availability of skilled critical care staff.” [SID 34] - “Generally but with a very junior workforce which outs a lot of pressure on senior staff.” [SID 70] |
| 1. Staffing gaps on shift | | | | | |
| Maintaining GPICs compliant nurse to patient ratios  Using supernumerary and/or non-clinical time to backfill gaps in shift | | | | 4  7  12 | - “No HCA funded on the night shifts.” [SID 92] - “Biggest challenge would be not having enough supernumerary runners on shift…some with ECMO/mechanical support our need for runners is important to ensure safe care.” [SID 129] - “…at times find the ratio's of 1:2 challenging with the acuity of patients and skill mix. We also have high number of side rooms in our units…this impacts on staffing and budget.” [SID 22] - “…what we cannot do, on any shift, is provide 50% of the workforce at post ICU course level. We simply do not have enough staff to maintain GPICS ratios with post ICU course staff.” [SID 64] - “Band 7 team are expected to backfill clinical shifts but also maintain the management side of the unit…” [SID 30] - “NIC/Supernumerary nurse always counted into numbers.” [SID 92] |
| 1. Impact of hospital pressures on ICU nurse staffing establishment | | | | | |
| Patient flow  Staff redeployment to wards | | | | 11  13 | - “Delayed discharges from ITU often impacting flow and pushing unit into escalation…” [SID 111] - “Other challenges include patient flow in the hospital. At times we struggle with flow and have delayed discharges because of this.” [SID 6] - “… when the nurse leads request staff to be moved to other wards to support short staffing elsewhere. Although there is no expectation to drop below the 1:1 nursing care level, there would potentially be an ask to redeploy the unit coordinator or ward managers, this reduces resources indirectly.” [SID 40] |
| 1. Staff vacancies, retention and recruitment | | | | | |
| Backfilling maternity leave | | | | 15  8 | - “Recruitment issues - at least 3-4 months to fill a post after someone leaves if not appointing internally…We have a pretty much open advert running all the time for band 5 staff in our intensive care units (unit 1 and 2).” [SID 110] - “…the challenges are when we have staff leave- giving 6 weeks notice but taking 3 months to have a replacement member of staff able to work in the team.” [SID 49] - “We have had a 10% maternity leave on 1 site which made staffing difficult.” [SID 13] - “difficult to cover fixed term maternity post” [SID 1] |
| 1. Unpredictability of admissions and fluctuations in patient need | | | | | |
|  | | | | 24 | - “Usually, challenges can be sharp fluctuations in the number of patient and their level of care.” [SID 19] - “we do have unpredictable admission due to the neuro speciality and aim to ensure we have the right ratio of staff to cover the empty beds. sometimes we have to double up patients in order to facilitate an admission…” [SID 17] |
| 1. Establishment calculation | | | | | |
|  | | | | 4 | - “Critical care uses many and varied high risk equipment and drugs but WFM only allows same headroom as ward staff.” [SID 107] - “Ability to admit with admitting staff is not included in establishment calculation yet an operational requirement.” [SID 53] |
| Q15b) Please describe the main safety and quality of care issues encountered in your critical care unit related to nurse staffing | | | | | |
| 1. Factors impacting quality of care | | | | | |
| Gaps in staffing and experience on shift  Staff wellbeing  Delays to patient flow  Junior skill mix  Maintaining staff and patient safety with unit geography  Patient characteristics  Diluted nurse to patient ratios | | | | 12  24  20  44  14  4  6 | - “The main issue is oversight and supervision at night at times of at or nearing total dependency.” [SID 121] - “Minimal number of HCAs, therefore staff having to leave their patient to collect equipment, answer phones, make/collect patient meals/refreshments reducing supervision.” [SID 40] - “Not always having a supernumerary nurse co-ordinator. Not having CSW over night…” [SID 11] - “High sickness post Covid - mental health / psychological support required- since reduced.” [SID 35] - “Staff health and well being - time management - late breaks and late documentation.” [SID 68] - “MSK injuries to staff - increased manual handling, increased acuity…” [SID 113] - “Getting timely discharges to enable us to do our work.” [SID 109] - “Delayed discharges impacting on effective utilisation of staffing as we use flexi rostering to ensure we have the right staff levels at the right time.” [SID 2] - “Junior skill mix is difficult to support on some shifts in very busy units.” [SID 21] - “Reduced skill mix in relation to the competency of staff after 2 years of little training/education.” [SID 38] - “Skill mix can affect the safety and quality of care, our aim is always to have 50% of staff rostered in possession of the ICU course.” [SID 82] - “Design of unit creates issues - never designed to have level 1 patients up and walking about next to level 3 acutely unwell patients …environment more than staffing has created challenges for aspects of safety.” [SID 110] - “… the environment as its old, very fragmented being split into 3-4 areas, poor line of sight and doesn't meet current HBN 04/02 standards.” [SID 37] - “Violence and aggression from delirious patients leading to staff injury.” [SID 113] - “challenging behaviour” [SID 20] - “When ratios are diluted we see an increase in incidents, staff struggle with workload and some aspects of care are missed. This is especially pertinent for long term patients, if we are unable to safely transfer them outside or mobilise in and out of bed.” [SID 122] |
| 1. Patient safety issues | | | | | |
| Pressure area care  Medication errors  Delays to and/or missed patient care  Infections  Falls | | | | 14  21  11  4  4 | - “Increased incidences of pressure ulcers…” [SID 18] - “…drug errors when staffing is tight and junior staff not adequately supported.” [SID 107] - “Medication errors seem to be more frequent (but not formally measured)” [SID 120] - “Potential delay in patient care while waiting for other staff (HCAs) to support patient repositioning /personal care.” [SID 40] - “…delay in implementing prescribed treatments ie. haemofiltration (rare, but staff statements tend to suggest the felt too busy)” [SID 68] - “Increased incidences of …hospital acquired infections” [SID 18] - “…blood stream infection, chest infection…” [SID 60] - “Supplementary care becoming more apparent throughout the trust and within critical care leading to an increased risk of falls.” [SID 126] |
| 1. Fundamentals of nursing care post-pandemic | | | | | |
|  | | | | 12 | - “Post pandemic we feel that we need to get 'back to basics' and drill down some of the fundamental basic components of nursing care practice. During the pandemic some elements of care were scaled down to cope with the insane workload.” [SID 6] - “Lack of awareness in risk or safe procedures, code of conduct or Policy despite receiving the appropriate training and assessments…” [SID 53] |
