# Supplementary material for: National survey evaluating the introduction of new and alternative staffing models in intensive care (SEISMIC-R) in the UK
Source: BMJ Open. 2025 Apr 10;15(4):e088233. doi: 10.1136/bmjopen-2024-088233 (PMC11987101; doi:10.1136/bmjopen-2024-088233)
Supplement: online supplemental file 2 [file bmjopen-15-4-s002.docx]

# Seismic-R Survey_Final

1. **Seismic-R Survey**

**SEISMIC-R Survey**

**This survey should take around 15 minutes to complete.  If you initially start the survey and need to complete at a later time, ensure that you access the survey link/code using the same device and your progress will be saved.**

**2. Information and Consent Sheet**

### **Dear Colleague, We would like to ask you to participate in this survey for the SEISMIC-R study (A Study to Evaluate the Introduction of new Staffing Models in Intensive Care: a Realist evaluation) which is surveying all 294 ICU units across the UK. The SEISMIC-R study is funded by the NIHR (Ref: 135168) and been approved by the Health Research Authority (Ref: 316667). What are we trying to understand? Having enough nurses to staff intensive care units (ICUs) is important for patient safety, quality care and staff wellbeing. There is national guidance for how many nurses should care for ICU patients. The number of nurses per patient and different skills of the nurses working in an ICU are together know as a ‘nurse staffing model’. Across ICUs in the UK , different ratios of qualified and unqualified nursing staff are being tried. For example, some hospitals use a high proportion of non-registered nurses and others a low proportion of ICU qualified nurses.. However, there is little research to understand what influences decisions to use different nurse staffing models or how different models affect patient, staff and service outcomes. As part of the larger SEISMIC-R study we aim to identify the key components of optimal nurse staffing models for deployment in ICU. What do I have to do? We ask you to answer 15 questions about your unit staffing models and how your staffing is configured. The questionnaire takes approximately 10-15 minutes to complete; it should be completed within two weeks of receiving this invitation. Your participation in this study is voluntary and any information you provide will be anonymised for any report so it cannot be traced back to you or your unit. Only the research team will have access to your data and all data will be held securely on ICNARC and University of Hertfordshire data servers in accordance with GDPR regulations. If you have any questions regarding this study or would like additional information please contact the SEISMIC-R team (seismic-r@herts.ac.uk or n.pattison@herts.ac.uk). By filling in this survey you indicate that you understand its purpose and consent to the use of the data as indicated above. Should you decide not to complete the survey, the data you have entered up to that point will be retained and used as outlined above. Thank you for your help. Professor Natalie Pattison (Chief Investigator: SEISMIC-R) ***

| I have been provided with information to understand the method and purpose of this study. | I agree to participate in the SEISMIC-R online survey | I am aware that I can withdraw at any time without this in any way affecting me. | I understand that my data is collected and registered confidentially, and that no unit or personal identifiable information will be reported |
| --- | --- | --- | --- |
| \|  \| \| --- \| | \|  \| \| --- \| | \|  \| \| --- \| | \|  \| \| --- \| |

### **Please provide your job role (and name if possible)**

|  |
| --- |

**4. Question 1**

### **Q1a) Which hospital/Trust are you based in?***

|  |  |
| --- | --- |
| Hospital Name | \|  \| \| --- \| |
| Trust Name | \|  \| \| --- \| |

### **Q1b) How many critical care (intensive care) units* does your hospital/Trust have? *critical care also includes those that are combined level 2/3 units, and HDU level 2 units.**

|  | 1 |
| --- | --- |
|  | 2 |
|  | 3 |
|  | 4 |
|  | 5 |
|  | 6 |

### **Q1c) How many beds does each unit have? (if more than 1 unit please continue to complete for each)**

| Unit 1 | \|  \| \| --- \| |
| --- | --- | --- |
| Unit 2 | \|  \| \| --- \| |
| Unit 3 | \|  \| \| --- \| |
| Unit 4 | \|  \| \| --- \| |
| Unit 5 | \|  \| \| --- \| |
| Unit 6 | \|  \| \| --- \| |

### **Q1d) Please describe the units**

|  | Unit Type |
| --- | --- |
| Unit 1 | \|  \| \| --- \| |
| Unit 2 | \|  \| \| --- \| |
| Unit 3 | \|  \| \| --- \| |
| Unit 4 | \|  \| \| --- \| |
| Unit 5 | \|  \| \| --- \| |
| Unit 6 | \|  \| \| --- \| |

**5. Question 2**

### **Q2a) How many level 2 beds does your unit/s have in total? (If more than one unit, please provide beds per unit).**

| Unit 1 | \|  \| \| --- \| |
| --- | --- | --- |
| Unit 2 | \|  \| \| --- \| |
| Unit 3 | \|  \| \| --- \| |
| Unit 4 | \|  \| \| --- \| |
| Unit 5 | \|  \| \| --- \| |
| Unit 6 | \|  \| \| --- \| |

### **Q2b) How many level 3 beds does your unit/s have in total? (If more than one unit, please provide beds per unit).**

| Unit 1 | \|  \| \| --- \| |
| --- | --- | --- |
| Unit 2 | \|  \| \| --- \| |
| Unit 3 | \|  \| \| --- \| |
| Unit 4 | \|  \| \| --- \| |
| Unit 5 | \|  \| \| --- \| |
| Unit 6 | \|  \| \| --- \| |

Please feel free to explain further, e.g. how much the level 2/3 bed mix fluctuates

|  |
| --- |

**6. Question 3**

### **Q3) What nurse model/s are you currently using in your critical care unit? (Please describe briefly, e.g. 1:1 for level 1 and 1:2 for level 2; proportions of RNAs/HCSWs, and if this differs for each unit indicated above)**

| Unit 1 | \|  \| \| --- \| |
| --- | --- | --- |
| Unit 2 | \|  \| \| --- \| |
| Unit 3 | \|  \| \| --- \| |
| Unit 4 | \|  \| \| --- \| |
| Unit 5 | \|  \| \| --- \| |
| Unit 6 | \|  \| \| --- \| |

**7. Question 4**

**Establishments**

### **Q4) What is the number of funded and approved Whole Time Equivalent (WTE) for registered nurses within each unit?**

| Unit 1 | \|  \| \| --- \| |
| --- | --- | --- |
| Unit 2 | \|  \| \| --- \| |
| Unit 3 | \|  \| \| --- \| |
| Unit 4 | \|  \| \| --- \| |
| Unit 5 | \|  \| \| --- \| |
| Unit 6 | \|  \| \| --- \| |

**8. Question 5**

### **Q5a) What is the funded and approved WTE for registered nurses in ICU actually in post in total across the organisation? ***

|  |
| --- |

### **Q5b) What is the funded and approved WTE for registered nurses actually in post in your unit/s? (If more than 1 unit, please continue to complete for each)**

| Unit 1 | \|  \| \| --- \| |
| --- | --- | --- |
| Unit 2 | \|  \| \| --- \| |
| Unit 3 | \|  \| \| --- \| |
| Unit 4 | \|  \| \| --- \| |
| Unit 5 | \|  \| \| --- \| |
| Unit 6 | \|  \| \| --- \| |

### **Q5c) What is the funded and approved WTE for non-registered nurses (including healthcare support workers and RNAs/TNAs) actually in post in your unit/s? (If more than 1 unit, please continue to complete for each)**

| Unit 1 | \|  \| \| --- \| |
| --- | --- | --- |
| Unit 2 | \|  \| \| --- \| |
| Unit 3 | \|  \| \| --- \| |
| Unit 4 | \|  \| \| --- \| |
| Unit 5 | \|  \| \| --- \| |
| Unit 6 | \|  \| \| --- \| |

### **Q5d) What is the funded and approved headcount (number of registered nurses, RNAs/TNAs and healthcare support workers) actually in post?**

| Unit 1 | \|  \| \| --- \| |
| --- | --- | --- |
| Unit 2 | \|  \| \| --- \| |
| Unit 3 | \|  \| \| --- \| |
| Unit 4 | \|  \| \| --- \| |
| Unit 5 | \|  \| \| --- \| |
| Unit 6 | \|  \| \| --- \| |

**9. Question 6**

### **Q6) What is the proportion of registered nurses with a post-registration qualification in your nursing establishment? ***

|  |
| --- |

**10. Question 7 and 8**

### **Q7) How are your daily ICU nurse (registered and non-registered) staffing requirements calculated?**

| Unit 1 | \|  \| \| --- \| |
| --- | --- | --- |
| Unit 2 | \|  \| \| --- \| |
| Unit 3 | \|  \| \| --- \| |
| Unit 4 | \|  \| \| --- \| |
| Unit 5 | \|  \| \| --- \| |
| Unit 6 | \|  \| \| --- \| |

**Q8) How are your total ICU nurse (registered and non-registered) staffing requirements calculated?
(e.g. 5.5 WTE nurses per bed)**

| Unit 1 | \|  \| \| --- \| |
| --- | --- | --- |
| Unit 2 | \|  \| \| --- \| |
| Unit 3 | \|  \| \| --- \| |
| Unit 4 | \|  \| \| --- \| |
| Unit 5 | \|  \| \| --- \| |
| Unit 6 | \|  \| \| --- \| |

**11. Question 9**

### **Q9a) Have ICU nurse staffing establishments changed since COVID-19?**

|  |  |
| --- | --- |
| Unit 1 | \|  \| \| --- \| |
| Unit 2 | \|  \| \| --- \| |
| Unit 3 | \|  \| \| --- \| |
| Unit 4 | \|  \| \| --- \| |
| Unit 5 | \|  \| \| --- \| |
| Unit 6 | \|  \| \| --- \| |

### **Q9b) If yes or not sure (e.g. you were not in this post during COVID), please provide more detail**

|  |
| --- |

**12. Question 10**

### **Q10) How has the way critical care nurses are allocated changed since the pandemic? (Please select all that apply)**

|  | Ratios of RNs to patients (increased) |
| --- | --- |
|  | Ratios of RNs to patients (decreased) |
|  | Numbers of critical care qualified nurses to patients (increased) |
|  | Numbers of critical care qualified nurses to patients (decreased) |
|  | Numbers of RNAs (increased) |
|  | Numbers of RNAs (decreased) |
|  | Increase in skill mix (more RNs) |
|  | Decrease in skill mix (less RNs) |
|  | Number of healthcare support workers (increased) |
|  | Number of healthcare support workers (decreased) |
|  | Other (please specify):   \|  \| \| --- \| |

**13. Question 11**

### **Q11a) Have there been any benefits (since 2020) of the current staffing model you are working in? (Please select all that apply)**

|  | Improved staff retention |
| --- | --- |
|  | Improved staff turnover |
|  | Better skill mix |
|  | Increased numbers of non-RNs |
|  | Improved flexibility in working patterns |
|  | Better nursing care |
|  | Improved sickness/absence rates |
|  | Decreased staffing costs |
|  | Other (please specify):   \|  \| \| --- \| |

### **Q11b) Have there been any disadvantages (since 2020) of the current staffing model you are working in? (Please select all that apply)**

|  | Worse staff retention |
| --- | --- |
|  | Worse staff turnover |
|  | Worse skill mix |
|  | Decreased numbers of non-RNs |
|  | Lack of flexibility in working patterns |
|  | Worse nursing care |
|  | Increased sickness/absence rates |
|  | Increased staffing costs |
|  | Other (please specify):   \|  \| \| --- \| |
|  |  |

**14. Question 12**

### **Q12a) Does your unit gather any of the following incident data and report this back to the unit? (please select all that apply)**

|  | Patient Safety Events | Staff Events |
| --- | --- | --- |
| Unplanned extubation |  |  |
| Vasopressor infusions running out |  |  |
| Accidental disconnection of arterial line |  |  |
| Accidental disconnection of central line |  |  |
| Patient falls |  |  |
| Infection rates |  |  |
| Pressure ulcer incidence/prevalence |  |  |
| Medication errors/incidents |  |  |
| Nurse sickness/absence rates |  |  |
| Staff retention/turnover |  |  |
| None of the above |  |  |

### **Q12b) Is any of the following data used to formally monitor quality of nursing care by nursing leadership? (i.e. to monitor the impact of nurse staffing on care quality). (please select all that apply)**

|  | Patient Safety Events | Staff Events |
| --- | --- | --- |
| Unplanned extubation |  |  |
| Vasopressor infusions running out |  |  |
| Accidental disconnection of arterial line |  |  |
| Accidental disconnection of central line |  |  |
| Patient falls |  |  |
| Infection rates |  |  |
| Pressure ulcer incidence/prevalence |  |  |
| Medication errors/incidents |  |  |
| Nurse sickness/absence rates |  |  |
| Staff retention/turnover |  |  |
| None of the above |  |  |

If so, please describe how

|  |
| --- |

**15. Question 13 and 14**

### **Q13) How do you think increased nurse staffing requirements should be managed in general? (not in surge situations, please describe).**

|  |
| --- |

### **Q14) Are there any changes you would like to see in how your staffing in your unit/s is managed? (please describe, e.g. skill mix adjustment)**

|  |
| --- |

**16. Question 15**

### **Q15a) Are you always able to deliver the planned staffing model and what challenges are there to it?**

|  |
| --- |

### **Q15b) Please describe the main safety and quality of care issues encountered in your critical care unit related to nurse staffing.**

|  |
| --- |
