# Supplementary material for: National survey evaluating the introduction of new and alternative staffing models in intensive care (SEISMIC-R) in the UK
Source: BMJ Open. 2025 Apr 10;15(4):e088233. doi: 10.1136/bmjopen-2024-088233 (PMC11987101; doi:10.1136/bmjopen-2024-088233)
Supplement: Supplementary file 3 [file bmjopen-2024-088233.reviewer_comments.pdf]

PEER REVIEW HISTORY

BMJ Open publishes all reviews undertaken for accepted manuscripts. Reviewers are asked to complete a checklist review form and are provided with free text boxes to elaborate on their assessment. These free text comments are reproduced below.

ARTICLE DETAILS

Title (Provisional)

A National Survey Evaluating the Introduction of new and alternative Staffing Models in Intensive Care (SEISMIC-R) in the UK

Authors

Hadley, Rebecca; DOGAN, Burcu; Wood, Niamh; Bohnacker, Niamh; Mouncey, Paul R.; Pattison, Natalie; SEISMIC-R Investigators, SEISMIC-R Investigators

---

VERSION 1 - REVIEW

**Reviewer**	**1**
**Name**	**Mohamed, Sherif Ahmed Abdelwahab**
**Affiliation**	**Assiut University**
**Date**	**10-Aug-2024**
**COI**	**No competing interests.**

Thank you very much for allowing me to review the manuscript entitled (A National Survey Evaluating the Introduction of New Staffing Models in Intensive Care (SEISMIC-R).

The study idea is interesting. The paper was well-written and organized.

Comment

In the discussion section, the authors should clarify their obtained results by comparing them with similarly published literature, explaining in details the similarities, differences, and arguments of the current study results with previous studies.

**Reviewer**	**2**
**Name**	**van der Jagt, Mathieu**
**Affiliation**	**Erasmus MC, Department of Intensive Care**
**Date**	**28-Aug-2024**
**COI**	**no**

The authors sought with a nationwide survey to assess ICU staffing by ICU nurses and changes related to the COVID pandemic.

This is important work, but readability is hampered in my view by unclear goals, main outcomes assessed and presentation of the main conclusions.

Some comments to improve:

- better explain all abbreviations throughout eg in text and legends to Tables and Figures (it hampers readability also)

- define the main outcomes of interest for this survey study in methods section, this will help align exact goals of the work with the results.

- Try to align better the conclusion with the main findings. I did not have the impression that the increase in non registered ICU nurses in the workforce since the covid pandemic was the most relevant finding to be mentioned in Abstract conclusion for instance. What about the bar graph with changes in nurse to patient ratios after COVID which is at least as relevant, although related. There are more significant findings that deserve highlighting in pertinent parts of the manuscript (see next comment).

- to this end, please make the First paragraph of Discussion a better reflection of all relevant main findings from this research, and make it a summary that clearly conveys these findings in a nutshell. It is now not very concrete what you found exactly. Same for Conclusion at the end of Discussion.

Reviewer 3

Name Hager, DN

Affiliation Division of Pulmonary and Critical Care Medicine, Johns Hopkins University School of Medicine, Medicine

Date 11-Nov-2024

COI None

General Comments:

1. Please number pages. Lack of page numbers makes it difficult to communicate feedback.

2. Paper is written with the assumption that the reader will know most abbreviations. It reads almost like and internal report from one hospital or governmental department. As we hope this will be read by a much broader population, the authors need to take time to better explain the UK system, provide a list of definitions, and better describe where this project falls within other ongoing projects with similar name.

3. Because of the writing, it is difficult to follow what I think could be important messages about the opportunities to support primary nurses with other types of workers to expand the capacity of busy ICUs.

4. I would better be able to critique the manuscript and the findings when the writing is improved. The topic is incredible important, the authors have access to very important data. However, as a non-UK reader, I'm lost quickly and the makes interpretation of the results and quality of the work difficult to judge.

Introduction:

1. Please spell use full names prior to introducing abbreviations. What is OECD for example?

2. Intro line 6. RNs "in post in" ICUs across England…. "in post in" may make sense in UK, but it struck me initially as poor grammar. Is there are different way to express this?

3. If this article will be read by people outside the UK who are not familiar with what Level 1, 2, and 3 patients indicate, it should be explained prior to using these terms.

4. Many readers will not understand the "Trust" concept. Please define.

5. You are using SEISMIC abbreviation prior to defining. I see it in the title, but help the reader.

6. When you describe what the SEISMIC study is, I think you should begin a new paragraph.

7. I see in the second paragraph, you define SEISMIC, but this really should come first. In the end, having read the paper twice, I'm still a little unclear if you are reanalyzing the SEISMIC data, or if you are doing something different from SEISMIC? It would be helpful to know with more clarity where your study fits within the SEISMIC organization.

Methods:

1. First paragraph: you mention changes since the covid pandemic, daylily and total nurse staffing establishments, changes to the establishments since covid and suggestions for alternative models. How are changes since the pandemic and changes to the establishment since covid different things. Please clarify. I think you are using descriptors not well known outside your work environment (or outside the UK) that are causing a lot of confusion.

2. Top of page 4 of the pdf: do you mean "domains" or "items" (1st word on page)? Looks like items. Are the "domains" those elements I highlight in #1 above?

3. Please include the survey as a supplement.

4. I would rephrase description of target population to be the persons that complete the survey and not the ICUs themselves. I would argue that what you call the target population is the "study environment" or "setting".

5. "Band"… another unfamiliar term.

6. How does it work to complete one survey for all ICUs in a hospital with multiple ICUs? It seems there is very likely to be different operational and staffing models in different ICUs. How would you navigate conflicting models from two ICUs in the same hospital?

7. How did you adjudicate which surveys to keep/discard. What is an eligible partial response? What is an unusable response? Who gets to decide?

Results:

1. As above, it is hard to understand how 196 surveys represent 300 units?

2. What is a “survey nominee”? Presuming this is the respondent, which would be a more traditional name I think.

3. PDF page 6 paragraph 3. You are giving some insight into what was happening in some specific units. Do I understand correctly that some units simply violated staffing ratio guidelines? Is that the point of this paragraph? If so, this seems like a very important finding and I’m interested to know what proportion of units did this? Do you know? Or, were these unusual occurrences?

4. Table 1 needs definitions detailed for all abbreviations.

5. PDF page 7, 1st paragraph under workforce model and characteristics…. “over establishment” means what. How is underfilled different than vacancy?

6. What is GPICSv2?

7. PDF page 7, paragraph under Post Covid 19 establishment change: You refer to “uplist in level 1, 3 and 4 beds, most frequently level 3”….. I don’t think you mean to refer to a level 4 bed, but the sentence if very unclear. What is an uplift. Please revise.

8. Table 2: suggest you simplify the table on remove the negatives. It will make reading the table easier. Providing the percent “no” in each category does not add information.

Discussion:

1. First paragraph should be a summary of what was done and major findings. Here, the authors are talking about prelim data (barriers sentence) and study strength. Study strength would be better ni a section on strengths and limitations.

2. PDF page 10, paragraph 1, line 3 – 5. An alternative reason for local attrition in the US was better compensation at other sites. Lots of people left the home site and became travelers (sometime just across town) to make a lot more money.

3. Same paragraph. Comment on perceived disadvantages. I would argue that greater investment in staffing (expenditure) may be a good thing.

4. PDF page 10, paragraph 3, first sentence. I think nurses suffer when objective measures of organ failure are used to determine RN:Pt ratios. A relatively stable patient on a ventilator may be much less work than a unintubated stable pt with a lot of questions or a family with

lots of questions. Too often, administrators equate organ dysfunction/illness severity to workload. In fact there is NO correlation between perceived workload and NPR (Hoonakker P, et al 2011) and it is perceived workload that is a predictor of poor pt outcomes (tubbs-cooley, jama peds, 2019, tubbs-cooley Intesn Crit Care Nurs 018).

VERSION 1 - AUTHOR RESPONSE

Reviewer: 1<br>Dr. Sherif Ahmed Abdelwahab Mohamed, Assiut University<br>Comments to the Author:<br>Thank you very much for allowing me to review the manuscript entitled (A National Survey Evaluating the Introduction of New Staffing Models in Intensive Care (SEISMIC-R).<br><br>The study idea is interesting. The paper was well-written and organized.<br>Comment<br>In the discussion section, the authors should clarify their obtained results by comparing them with similarly published literature, explaining in details the similarities, differences, and arguments of the current study results with previous studies.<br><br>--<br>Reviewer: 2<br>Dr. Mathieu van der Jagt, Erasmus MC<br>Comments to the Author:<br>The authors sought with a nationwide survey to assess ICU staffing by ICU nurses and changes related to the COVID pandemic. This is important work, but readability is hampered in my view by unclear goals, main outcomes assessed and presentation of the main conclusions.<br><br>Some comments to improve:<br>- better explain all abbreviations throughout	Reviewer 1<br><br>Thank you for the kind and positive comments. We have now revised the discussion in light of the feedback and in relation to recent literature. We have added in further comparative literature in the discussion: lines X and X<br><br>“A recent study in Australia highlighted how lower levels of critical care registered nurses (CCRNs) was directly linked to mortality. Units with 50-75% CCRNs has higher patient mortality risk than those with more than 75% CCRNs (adjusted OR 1.21 [95% CI 1.02-1.45]) (Ross et al, 2024).”<br><br>Using acuity in this way risks undermining nursing workload[1], and using patient-based workload like acuity poorly correlated with perceived ICU workload[2]. Nursing dependency is managed through professional judgment used alongside nationally guided ratios[3], as evidenced in this study. Research in NICU has also found that subjective view of workload are associated with missed care[4], demonstrating the importance of workload perceptions for patient outcomes.<br><br>---<br><br>Reviewer 2<br><br>Thank you for taking the time to review. We have revised the text throughout, including better explanation of UK-centric which we believe improves the manuscript.

eg in text and legends to Tables and Figures (it hampers readability also)	Apologies, we have amended this throughout all tables.
- define the main outcomes of interest for this survey study in methods section, this will help align exact goals of the work with the results.	Thank you for your suggestion. We have now added in a sentence below the aim on expected outcomes.
Try to align better the conclusion with the main findings. I did not have the impression that the increase in non registered ICU nurses in the workforce since the covid pandemic was the most relevant finding to be mentioned in Abstract conclusion for instance. What about the bar graph with changes in nurse to patient ratios after COVID which is at least as relevant, although related. There are more significant findings that deserve highlighting in pertinent parts of the manuscript (see next comment).<br>- to this end, please make the First paragraph of Discussion a better reflection of all relevant main findings from this research, and make it a summary that clearly conveys these findings in a nutshell. It is now not very concrete what you found exactly. Same for Conclusion at the end of Discussion.	Thank you for your suggestion. We have revised the discussion to better summarise the research and revised conclusion as outlined below and woven in additional evidence, although the evidence remains limited.
---	---
Reviewer: 3<br>Dr. DN Hager, Division of Pulmonary and Critical Care Medicine, Johns Hopkins University School of Medicine<br>Comments to the Author:<br>General Comments:	We thank the reviewer for taking the time to review our research.
1. Please number pages. Lack of page numbers makes it difficult to communicate feedback.	1.We have added in page numbers – page numbers were included on the pdf.
2. Paper is written with the assumption that the reader will know most abbreviations. It reads almost like and internal report from one hospital or governmental department. As we hope this will be read by a much broader population, the authors need to take time to better explain the UK system, provide a list of definitions, and better describe where this project falls within other ongoing projects with similar name.	2. We have revised the abbreviations throughout, and we apologise for this oversight, and have better described the work to make this less UK-focused, and provide UK context. We have also included a description of the SEISMIC work (the smaller, precursor to this study). We have also provided a description of the function of certain organisations like ICNARC.

3. Because of the writing, it is difficult to follow what I think could be important messages about the opportunities to support primary nurses with other types of workers to expand the capacity of busy ICUs.<br>4. I would better be able to critique the manuscript and the findings when the writing is improved. The topic is incredible important, the authors have access to very important data. However, as a non-UK reader, I'm lost quickly and the makes interpretation of the results and quality of the work difficult to judge.<br><br>Introduction:<br>1. Please spell use full names prior to introducing abbreviations. What is OECD for example?<br><br>2. Intro line 6. RNs "in post in" ICUs across England…. "in post in" may make sense in UK, but it struck me initially as poor grammar. Is there are different way to express this?<br><br>3. If this article will be read by people outside the UK who are not familiar with what Level 1, 2, and 3 patients indicate, it should be explained prior to using these terms.	3. Thank you for the feedback. We have revised throughout including making the key messages clearer (please see discussion section below).<br><br>1.This is now in full, but is an accepted abbreviation in international journals, including BMJ and BMJ Open, as it is so widely referenced across many spheres as an accepted acronym.<br><br>2.We have now revised this.<br><br>3.Thank you we have now revised this, and added in the following:<br><br>"Level 1 is enhanced care, for patients requiring a higher level of monitoring but not critical care, level 2 care is for those needing two or more basic organ system monitoring/support, or single organ support at an advanced level (other than advanced respiratory support), or long term advanced respiratory support, or high levels of nursing dependency not able to be provided in a level 1 area. Level 3 care involves advanced respiratory support, or monitoring/support for two or more organ systems at an advanced level. It also includes level 2 patents with delirium/agitation or those with chronic impairment of at least one organ that restricts daily activities (co-morbidity) and who require support for an acute reversible failure of another organ system."<br><br>4.Thank you this had been revised (see page X line X) and we have revised to:<br>"Private ICUs, Paediatric ICUs, and HDUs were excluded from the sample, resulting in a target population of 331 units (across 231 hospitals/155 trusts). Hospitals in the UK sit within wider acute NHS Trusts, so frequently comprise several

4. Many readers will not understand the “Trust” concept. Please define.	hospitals, and several ICUs, which may be managed differently, or independently even within the same NHS Trust.”
	5.Thank you for picking this up, this has now been revised.
5. You are using SEISMIC abbreviation prior to defining. I see it in the title, but help the reader.	6. Thank you, this has been revised.
6. When you describe what the SEISMIC study is, I think you should begin a new paragraph.<br>7. I see in the second paragraph, you define SEISMIC, but this really should come first. In the end, having read the paper twice, I’m still a little unclear if you are reanalyzing the SEISMIC data, or if you are doing something different from SEISMIC? It would be helpful to know with more clarity where your study fits within the SEISMIC organization.	7.Revised to make this clearer; this is not a secondary analysis but entirely new data. This is also clarified page X line X…
	Methods
Methods:<br>1. First paragraph: you mention changes since the covid pandemic, daylily and total nurse staffing establishments, changes to the establishments since covid and suggestions for alternative models. How are changes since the pandemic and changes to the establishment since covid different things. Please clarify. I think you are using descriptors not well known outside your work environment (or outside the UK) that are causing a lot of confusion.	1.Thank you for your suggestions. We have clarified what establishment, and other relevant descriptors noted by reviewers, relate to. In our aim we sought to understand what the establishment changes were as part of the survey, alongside another other key staffing - related changes the respondents wished to report, which previously had only been reported anecdotally<br><br>*“Specifically, we report here on the results of a national survey of ICUs intended to establish the organisation of nurse staffing models, to provide an understanding of models in use and any changes to staffing models and practices since COVID-19.”*
2. Top of page 4 of the pdf: do you mean “domains” or “items” (1st word on page)? Looks like items. Are the “domains” those elements I highlight in #1 above?	2. We did intend to mean items (survey items), as stated. We apologise but we could not see reference to the word domains in the manuscript?
3. Please include the survey as a supplement.	3.Thank you we have now included this.
	4.Thank you for this suggestion. The target population was the most senior nursing

4. I would rephrase description of target population to be the persons that complete the survey and not the ICUs themselves. I would argue that what you call the target population is the "study environment" or "setting".	representative from each of the National Health Service (NHS) state-funded, not private) adult ICUs across the UK
5. "Band"… another unfamiliar term.	5. We have added in description of this. 'band 5s are junior nurses, the lowest grade of registered nurse in the UK, comprising the largest portion of the UK workforce – up to band 8s who are the most senior nurse leaders in ICU' (line x page X)
6. How does it work to complete one survey for all ICUs in a hospital with multiple ICUs? It seems there is very likely to be different operational and staffing models in different ICUs. How would you navigate conflicting models from two ICUs in the same hospital?	6. Some matrons (lead nurses) manage all the ICUs within a Trust (incorporating several ICUs), so were able to provide information for each ICU, each of which were inputted discretely (which we hope is clearer with the addition of the full questionnaire in a supplemental file). "Where the lead nurse delegated to hospital (ICU) leads, we analysed results for each, as above. We looked at each data entry separately in terms reported models." But we do agree this is a limitation of the survey which we have reflected on in the discussion. We have also added in the following: "Where models differed within a Trust, we asked for detail on this in open-ended data".
7. How did you adjudicate which surveys to keep/discard. What is an eligible partial response? What is an unusable response? Who gets to decide?	7. In regard to choosing which to disregard, we (*a priori*) determined a minimum level of data to be reported including, number of beds, model in use, number of nurses (establishment). We found that a few respondents started to complete the survey but did not get further than naming the hospital or reporting on beds, so no meaningful data regarding staffing was yielded.
Results:<br>1. As above, it is hard to understand how 196 surveys represent 300 units?	Results<br>1.This is now made more clear with the explanation of hospital trusts in the UK, which routinely incorporate several ICUs, all of which may be managed differently, or independently even within the same NHS Trust. (line X page X)
2. What is a "survey nominee"? Presuming this is the respondent, which would be a more traditional name I think.	2.We have clarified this in the text – as the right person needed to complete the survey (not just a senior nurse manager, but the overarching
3. PDF page 6 paragraph 3. You are giving some insight into what was happening in	

some specific units. Do I understand correctly that some units simply violated staffing ratio guidelines? Is that the point of this paragraph? If so, this seems like a very important finding and I'm interested to know what proportion of units did this? Do you know? Or, were these unusual occurrences?	manager/unit head for the unit, or person nominated by unit head). We have used both terms as one indicates intended recipient and the other, respondent.<br><br>3. Yes, one unit did violate and yes this is the point of this paragraph. However, it was only 1 unit that elected to report this in the open-ended data, so a very low proportion overall, but we wanted to report this, just as the reviewer indicated we also felt this was important to share as other units may have experienced this but not chosen to report it. We have also now picked this up in the discussion. "We relied on staff to report difference in models and also describe in more detail in open-ended data; there could have been further variation in practices not reported beyond the simplistic adherence to guidance."
4. Table 1 needs definitions detailed for all abbreviations.	4. we have amended this, thank you.
5. PDF page 7, 1st paragraph under workforce model and characteristics…. "over establishment" means what. How is underfilled different than vacancy?	5. we have now clarified this, thank you.
6. What is GPICSv2?	6.We defined this when first used on page X, line X. GPICS v2 is national guidance (Guidelines for the Provision of Intensive Care Services version 2[GPICSv2]).
7. PDF page 7, paragraph under Post Covid 19 establishment change: You refer to "uplist in level 1, 3 and 4 beds, most frequently level 3"….. I don't think you mean to refer to a level 4 bed, but the sentence if very unclear. What is an uplift. Please revise.	7. Thank you, we have reworded uplift. We do mean level 4 beds (which are highly specialist ECMO beds – and have clarified this).
8. Table 2: suggest you simplify the table on remove the negatives. It will make reading the table easier. Providing the percent "no" in each category does not add information.	8. Thank you, we have revised the table for ease of reading as suggested
Discussion:<br>1. First paragraph should be a summary of what was done and major findings. Here, the authors are talking about prelim data (barriers sentence) and study strength. Study strength would be better ni a section on strengths and limitations.	1.Thank you for the suggestion. We have restructured the discussion so it read more clearly, and followed the reviewer 3's suggestion here, which also addresses reviewer 2's comment.
2. PDF page 10, paragraph 1, line 3 – 5. An alternative reason for local attrition in the US was better compensation at other sites. Lots of people left the home site and became	2.Thank you for raising this. However, this interesting phenomenon raised by reviewer 3 of

travelers (sometime just across town) to make a lot more money.<br><br>3. Same paragraph. Comment on perceived disadvantages. I would argue that greater investment in staffing (expenditure) may be a good thing.<br><br>4. PDF page 10, paragraph 3, first sentence. I think nurses suffer when objective measures of organ failure are used to determine RN:Pt ratios. A relatively stable patient on a ventilator may be much less work than a unintubated stable pt with a lot of questions or a family with lots of questions. Too often, administrators equate organ dysfunction/illness severity to workload. In fact there is NO correlation between perceived workload and NPR (Hoonakker P, et al 2011) and it is perceived workload that is a predictor of poor pt outcomes (tubbs-cooley, jama peds, 2019, tubbs-cooley Intesn Crit Care Nurs 018).	nurses leaving their home hospital for better pay did not occur in the UK. The pay banding is a national system so rates of pay are set by government not hospitals. Nurses did not move to gain higher pay rates during the pandemic. Given this is not reported in any international literature that we could find, the authors feel it would be difficult to weave this in without substantiation. We have also provided corroborating evidence to support our assertion.<br><br>3. thank you for this suggestion. We have added in context and clarified this – separating it out form disadvantages: 'As a broader benefit, there was an increased expenditure on staffing (25.5%), however open-ended data indicated that nurse leaders also saw this as a new added pressure.'<br><br>4. Thank you, we agree with this sentiment and have added in wording to acknowledge this helpful suggestion.<br><br>" Using acuity in this way risks undermining nursing workload[1], and using patient-based workload like acuity poorly correlated with perceived ICU workload[2]. Nursing dependency is managed through professional judgment used alongside nationally guided ratios[3], as evidenced in this study. Research in NICU has also found that subjective view of workload are associated with missed care[4], demonstrating the importance of workload perceptions for patient outcomes. "

VERSION 2 - REVIEW

Reviewer 3

Name Hager, DN

Affiliation Division of Pulmonary and Critical Care Medicine, Johns Hopkins University School of Medicine, Medicine

Date **20-Jan-2025**

COI

General Comments:

Thank you for allowing me to review this new draft of the manuscript. I see you have tried earnestly to respond to prior comments. The topic remains important and one of interest to me. However, I still find the writing very difficult to follow. I do have a better understanding of the environment than from the first draft. Thank you. I did note in review of the survey tool itself, that Reponses were likely to be answered inconsistently by different respondents who were at risk of interpreting questions in ways they were not intended to be interpreted. Was there an operations manual provided to respondents to facilitate understanding? This really challenges one to feel they are interpreting data well and gives me uncertainty about the findings reported.

Specific Comments:

Abstract

1. Participant are the people completing the survey, not the individual units.

Introduction

1. Page 2, Lines 40 – 50: This description is helpful, but a distraction in the introduction where I am hoping to learn what your study is about and why it is needed. This description of the UK system would be better in a methods section or in a supplement.

2. The first paragraph should be broken up in the 2 – 3 paragraphs.

3. Page 2 Line59 – 60. Your parentheses are not well places.

4. Page 2 Line 58: What do you mean by “account”…. Do you mean “take advantage of, allow, utilize”? Seems account is not the best word choice.

5. Page 2, Line 57: do you mean increased “capacity” or “demand”.

6. Page 3, Line 7: what does “realist” mean here? I think not all readers will be familiar with the concept of a “realist review”.

7. Page 3, Line 6: over pandemic or during pandemic.

8. Page 3, Lines 10 – 17: Why are you reviewing results of the earlier SEISMIC study here. Seams this is not motivation of the current work. These thoughts, seem more relevant to a discussion section.

9. Page 3, Line 24-25: you are not characterizing the “impact” of different models on “patient outcomes”. You have no patient data.

Methods

1. Your survey appears to be closer to 26 items.

2. Page 3, line 58: appear to have extra parentheses in line.

3. Page 4, Line 9: you are referring to ICU or Units as a "population". I think what you mean is that you are trying to represent operations in 331 units. This is different than a population of interest.

4. Page 4, Line9 – 11: You say "Hospitals in the UK sit within wider acute NHS Trusts, so frequently comprise several hospitals, and several ICUs, which may be managed differently, or independently even withing the sam NHS trust." What this actually says is "Hospitals frequently comprise several hospitals…". I don't think this is what you mean. Please revise so the meaning is clear. Do you mean that NHS Trusts can have several hospitals within them?

Results

1. Page 5 paragraph 1: This presentation of data is a little confusing. Would suggest you simply say that 162 discrete surveys were retained after duplicates and partial responses were excluded leading to a response rate of 70.1%. Then say this represented x units representing y different trusts. The 90.6% and 83.9% don't really help the reader if many of those surveys were discarded.

2. Page 5 paragraph 1: do the 162 discrete surveys still describe 300 ICUs in 130 trusts? If not, please adjust these numbers to reflect the ICUs and trusts the analyzed surveys (the 162 retained) represent.

3. Page 5 paragraph 2: The heading says "ICU unit characteristics". This paragraph speaks to who responded. It fist better with the previous heading.

4. Page 6 Paragraph 1, line 43: You say some units flex all bed to level three, and/or exceeding their funded level three….. what is meant hear by and/or?

5. Page 6, paragraph 1, line48 -49: I would think staff having to move between sites would be a credentialing challenge and a real dissatisfier. Do you mean between sites or between ICUs within a site?

6. Page 7, paragraph 1: it is a little difficulty to understand the meaning of WTE per bed when there are a mix of level 2 and level 3 beds. How are you navigating this issue when you report WTE/bed?

7. Page 7, paragraph 2: The proportion….. you then site 48.9 and a SD. Is this percent? The parenthetical statement "(with reported variation in interpretation drawn from open-ended contextual data)" makes no sense to me. What does this mean?

8. General comment: you often open parenthetical statements, but don't close them.

9. Page 8, paragraph 1, line 13. You have previously presented data as N = 14/141 and a percentage. Why are you changing you approach here. It is a minor point, but consistency helps readers follow.

10. Page 8, Paragraph 2: you refer to “improved staff retention” and “improved staff turnover”. These seem directionally opposite. Improved/increased turnover would be a bad thing. Later, you flip it. It also seems redundant and will be a source of confusion to quantify both. I think the message gets through by speaking to staff retention OR staff turnover and not both. In the next paragraph you are talking about disadvantages related to attrition and retention? Pick one or be clear on how these things are different and provide clear directionality, what is good and what is bad.

11. Page 9, paragraph 1, last sentence. Point and meaning of this sentence is not clear. I don’t think you measured nursing care quality. You had mentioned pressure ulcers in the first sentence. Are you saying that the prevalence of pressure ulcers is a surrogate for nursing quality and as such was used as a surrogate of staffing compliance (does this mean adequate ratio) and to support workforce review (this says quality data facilitates the review process), staffing business cases (do you mean quality/safety data is used to support business plans for more support)….. Please see if you can construct this in a more clear manner.

Discussion

1. Page 9, second to last paragraph, line 51-52: what is 10.6% and what is 18.4%. It currently reads that nursing ratios are stable (only 10.6%) of the time and the numbers of qualified nurse to patients have decreased 18.4% of the time.

2. Page 9, last paragraph. Is only 24% of respondents reporting improved retention a good number? Is this down from a higher number?

3. Page 9 – 10: retention/turnover discussion. It seems you are trying to say that both turnover and retention have gotten better and worse. It seems this may be true in some places and not true in others. I think you need a better description of these data and interpretation in the discussion. See comments above.

4. Page 10, paragraph 3: “these contrasting results”? The ones cited in the prior paragraph all seem to be aligned.

VERSION 2 - AUTHOR RESPONSE

Response to Reviewer 3 comments

R3: I did note in review of the survey tool itself, that Reponses were likely to be answered inconsistently by different respondents who were at risk of interpreting questions in ways they were not intended to be interpreted. Was there an operations manual provided to respondents to facilitate understanding? This really challenges one to feel they are interpreting data well and gives me uncertainty about the findings reported.

We kindly thank Reviewer 3 for taking the time to provide an in-depth review on our revision. We have reviewed throughout as suggested by this reviewer and are confident this is now much clearer to readers. While we did not provide an explanatory manual, we did undertake face/content validity testing and piloted the questionnaire, and we fully acknowledge the limitations noted in our discussion.

Specific Comments:

Abstract
1. Participant are the people completing the survey, not the individual units.

Thank you we have amended this

Introduction
1. Page 2, Lines 40 – 50: This description is helpful, but a distraction in the introduction where I am hoping to learn what your study is about and why it is needed. This description of the UK system would be better in a methods section or in a supplement.

Thank you, we had included it here based on previous reviewers' comments, but have moved this to the methods section.

2. The first paragraph should be broken up in the 2 – 3 paragraphs.

Thank you, we have now broken this up.

3. Page 2 Line59 – 60. Your parentheses are not well places.

We have amended this, thank you for picking this up.

4. Page 2 Line 58: What do you mean by "account"…. Do you mean "take advantage of, allow, utilize"? Seems account is not the best word choice.

We have removed the word account, and reworded to: "The need for models that permit local variation, and allow reporting against, in skill"

5. Page 2, Line 57: do you mean increased "capacity" or "demand". We have amended to demand.

We have reworded to demand

6. Page 3, Line 7: what does "realist" mean here? I think not all readers will be familiar with the concept of a "realist review".

We have added in a few words to explain realist evaluation. Realist evaluation studies aim to provide programme theories to explain changes, outlining what works, for whom and in what circumstances.

7. Page 3, Line 6: over pandemic or during pandemic.

Thank you we have amended this.

8. Page 3, Lines 10 – 17: Why are you reviewing results of the earlier SEISMIC study here. Seams this is not motivation of the current work. These thoughts, seem more relevant to a discussion section.

We have moved some of this paragraph to the discussion, but retained reference to the SEISMIC work was the funded precursor and driver for this larger-scale piece of work within the same programme, so we do feel some of this explanation remains well-placed here.

9. Page 3, Line 24-25: you are not characterizing the "impact" of different models on "patient outcomes". You have no patient data.

We respectfully wish to retain this. We make it clear that this is part of a larger study which does report our large-scale patient data separately, as this is the overarching research Q for the study.
"Specifically, we report here on the results of a national survey of ICUs intended to establish the organisation of nurse staffing models, to provide an understanding of models in use and any changes to staffing models and practices since COVID-19."

This aim in itself (pertaining to the survey alone) outlines how we aimed to provide important data on the impact of different models, with that data ultimately feeding into the programme theories and being integrated with our large-scale modelling data.

Methods
1. Your survey appears to be closer to 26 items.

Thank you. We have now clarified the number of items and sub-items to make this clearer. Not all items were relevant to each respondent.

2. Page 3, line 58: appear to have extra parentheses in line.

Thank you we have amended

3. Page 4, Line 9: you are referring to ICU or Units as a "population". I think what you mean is that you are trying to represent operations in 331 units. This is different than a population of interest.

Thank you we have amended to sample

4. Page 4, Line9 – 11: You say "Hospitals in the UK sit within wider acute NHS Trusts, so frequently comprise several hospitals, and several ICUs, which may be managed differently, or independently even withing the sam NHS trust." What this actually says is "Hospitals frequently comprise several hospitals…". I don't think this is what you mean. Please revise so the meaning is clear. Do you mean that NHS Trusts can have several hospitals within them?

Thank you we have amended

Results

1. Page 5 paragraph 1: This presentation of data is a little confusing. Would suggest you simply say that 162 discrete surveys were retained after duplicates and partial responses were excluded leading to a response rate of 70.1%. Then say this represented x units representing y different trusts. The 90.6% and 83.9% don't really help the reader if many of those surveys were discarded.

We wanted to show coverage of ICU data, but appreciate this is confusing so have reworded this as suggested.

"A total of 196 responses were received for 300/331 units (representing 155 Trusts and 231 hospitals). 162 of these surveys were retained; Nine duplicates were identified, and 25 partial responses which did not meet the required minimum data were removed, leading to a total 162 individual responses from 231 hospitals for analysis, and a final response rate of 70.1%."

2. Page 5 paragraph 1: do the 162 discrete surveys still describe 300 ICUs in 130 trusts? If not, please adjust these numbers to reflect the ICUs and trusts the analyzed surveys (the 162 retained) represent.

Please see above.

3. Page 5 paragraph 2: The heading says "ICU unit characteristics". This paragraph speaks to who responded. It fist better with the previous heading.

Thank you we have amended

4. Page 6 Paragraph 1, line 43: You say some units flex all bed to level three, and/or exceeding their funded level three….. what is meant hear by and/or?

Thank you we have amended

5. Page 6, paragraph 1, line48 -49: I would think staff having to move between sites would be a credentialing challenge and a real dissatisfier. Do you mean between sites or between ICUs within a site?

We mean both between sites (travelling across cities) within the same Trust and between ICUs in the same hospital site. We have added this in to clarify. "(between ICUs on the same hospital site and across the city to ICUs within the same Trust)"

6. Page 7, paragraph 1: it is a little difficulty to understand the meaning of WTE per bed when there are a mix of level 2 and level 3 beds. How are you navigating this issue when you report WTE/bed?

This is an ongoing issue encountered by unit leads for national reporting and we have added in to clarify:

"Establishment is predicated on a nominally 'fixed' number of beds (usually around 75% level 3 beds and 25% level 2 beds, reviewed yearly for each unit by commissioners)."

7. Page 7, paragraph 2: The proportion….. you then site 48.9 and a SD. Is this percent? The parenthetical statement "(with reported variation in interpretation drawn from open-ended contextual data)" makes no sense to me. What does this mean?

Apologies for this oversight in reporting, we have amended this.

8. General comment: you often open parenthetical statements, but don't close them.

Apologies for this oversight, we have amended.

9. Page 8, paragraph 1, line 13. You have previously presented data as N = 14/141 and a percentage. Why are you changing you approach here. It is a minor point, but consistency helps readers follow.

Apologies, we have amended this.

10. Page 8, Paragraph 2: you refer to "improved staff retention" and "improved staff turnover". These seem directionally opposite. Improved/increased turnover would be a bad thing. Later, you flip it. It also seems redundant and will be a source of confusion to quantify both. I think the message gets through by speaking to staff retention OR staff turnover and not both. In the next paragraph you are talking about disadvantages related to attrition and retention? Pick one or be clear on how these things are different and provide clear directionality, what is good and what is bad.

Thank you- we had included both, but this is a fair comment. We have now amended this. The two questions generated different responses across the data and even contradiction within the same case for two respondents (and the free-text helped to explain some of this). We have now added this into limitations and added this in to clarify turnover: "rates of staff turnover (the proportion of nurses leaving each year)"**. Retention may also include those staying within ICU but in a different role, hence these are reported separately in the literature (see e.g. A systematic review of registered nurse turnover and retention in the United States. Woodward, Kyla F. et al. Nursing Outlook, Volume 70, Issue 4, 664 – 678).**

11. Page 9, paragraph 1, last sentence. Point and meaning of this sentence is not clear. I don't think you measured nursing care quality. You had mentioned pressure ulcers in the first sentence. Are you saying that the prevalence of pressure ulcers is a surrogate for nursing quality and as such was used as a surrogate of staffing compliance (does this mean adequate ratio) and to support workforce review (this says quality data facilitates the review process), staffing business cases (do you mean quality/safety data is used to support business plans for more support)….. Please see if you can construct this in a more clear manner.

We agree, we measured only those indicators nurse leaders used to show nursing care quality, rather than measuring care quality. We have hopefully clarified this better. "how data were used

to report against” quality of nursing care. . . The quality of nursing care data “was used by nurse leaders” to report on patient safety incidents. . .

Discussion

1. Page 9, second to last paragraph, line 51-52: what is 10.6% and what is 18.4%. It currently reads that nursing ratios are stable (only 10.6%) of the time and the numbers of qualified nurse to patients have decreased 18.4% of the time.

Thank you we have clarified by adding: “for more units (18.4%) than where it increased (10.6%), showing that while for some units ratios had improved, for a greater number it had worsened.”

2. Page 9, last paragraph. Is only 24% of respondents reporting improved retention a good number? Is this down from a higher number?

We have reworked this section to attempt to explain this better. This is not down from a higher number, as this was not a comparative survey, and we hope we have drawn out the nuance in the following:

“The most significant benefit of staffing models currently in use was perceived improved flexibility of staffing patterns (n=43, 30.5%) since the COVID-19 pandemic. And while improved staff retention (n=34, 24.1%) was reported by some, a larger number reported worsening turnover and retention was also reported, indicating a mixed picture, with some form of stabilisation of the workforce in only some units. The high attrition associated with the COVID-19 pandemic may have had a legacy impact, potentially because of high levels of burnout and significant impact on staff wellbeing and job satisfaction[21-23]. Staff retention and turnover are distinct but related concepts in the literature[24]. A key difference is that retention can indicate retained employment in the ICU, but possibly in a different position (e.g. non-direct care delivery role). With the advent of newer roles, like family-liaison nurse roles in ICU, this might have an effect on how nurse leaders responded to these linked questions.”

3. Page 9 – 10: retention/turnover discussion. It seems you are trying to say that both turnover and retention have gotten better and worse. It seems this may be true in some places and not true in others. I think you need a better description of these data and interpretation in the discussion. See comments above.

We agree this needed amending, thank you for the suggestion. We have also added in a comment in the limitations regarding bidirectionality of the survey questions.

We have amended and added to this paragraph (as outlined above):

4. Page 10, paragraph 3: “these contrasting results”? The ones cited in the prior paragraph all seem to be aligned.

We have amended this to avoid confusion.
